# Space‐Confined Amplification for In Situ Imaging of Single Nucleic Acid and Single Pathogen on Biological Samples

**DOI:** 10.1002/advs.202407055

**Published:** 2024-10-07

**Authors:** Tao Yang, Dong Li, Zisheng Luo, Jingjing Wang, Fangbin Xiao, Yanqun Xu, Xingyu Lin

**Affiliations:** ^1^ College of Biosystems Engineering and Food Science Zhejiang University Hangzhou 310058 China; ^2^ The Rural Development Academy Zhejiang University Hangzhou 310058 China; ^3^ State Key Laboratory of Fluid Power and Mechatronic Systems Zhejiang University Hangzhou 310058 China

**Keywords:** in situ detection, LAMP, space‐confined interfacial amplification, spatial imaging

## Abstract

Direct in situ imaging of nucleic acids on biological samples is advantageous for rapid analysis without DNA extraction. However, traditional nucleic acid amplification in aqueous solutions tends to lose spatial information because of the high mobility of molecules. Similar to a cellular matrix, hydrogels with biomimetic 3D nanoconfined spaces can limit the free diffusion of nucleic acids, thereby allowing for ultrafast in situ enzymatic reactions. In this study, hydrogel‐based in situ space‐confined interfacial amplification (iSCIA) is developed for direct imaging of single nucleic acid and single pathogen on biological samples without formaldehyde fixation. With a polyethylene glycol hydrogel coating, nucleic acids on the sample are nanoconfined with restricted movement, while in situ amplification can be successfully performed. As a result, the nucleic acids are lighted‐up on the large‐scale surface in 20 min, with a detection limit as low as 1 copy/10 cm^2^. Multiplex imaging with a deep learning model is also established to automatically analyze multiple targets. Furthermore, the iSCIA imaging of pathogens on plant leaves and food is successfully used to monitor plant health and food safety. The proposed technique, a rapid and flexible system for in situ imaging, has great potential for food, environmental, and clinical applications.

## Graphical abstract

Inspired by the nanoconfined spaces inside cells, in situ space‐confined interfacial amplification (iSCIA) is developed in hydrogels for the spatial imaging of single nucleic acid and single pathogen on biological samples. Using a powerful in situ‐confined enzymatic reaction, the hydrogel iSCIA can be applied for plant health self‐monitoring and food pathogen safety surveillance.

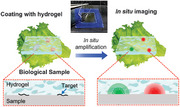

## 1 Introduction

Since the worldwide outbreak of diseases caused by diverse pathogens, such as viruses, bacteria, and fungi, nucleic acid testing with low time‐to‐result has played an important role in the in‐field detection of biohazard risks.^[^ ^1^ ^]^ Direct in situ imaging of nucleic acids on solid‐state biochemical samples (e.g., plants, food, and environment) is essential for identifying threats before pathogen spread, which is crucial for public health screening, plant health monitoring, and food safety surveillance.^[^ ^1^, ^2^ ^]^ Traditional nucleic acid enzymatic amplification test for a solid‐state sample requires swabbing steps to collect target from surface or performing DNA/RNA extraction, which is labor‐intensive and time‐consuming.^[^ ^3^ ^]^ Furthermore, due to the high mobility of molecules in aqueous solutions, conventional enzymatic amplification reactions cannot provide spatial information, and their original distribution is completely lost.

Recently, considerable attention has been paid to in situ amplification methods for the direct nucleic acid imaging of samples, such as fluorescence in situ hybridization (FISH), in situ polymerase chain reaction (PCR), and in situ rolling circle amplification (RCA).^[^ ^1^, ^4^ ^]^ However, all these methods require fixation of cells and nucleic acids using harsh chemical agents (such as paraformaldehyde), which takes several days to complete.^[^ ^4^, ^5^ ^]^ These harsh agents strongly inhibit nucleic acid amplification and should be removed before amplification. Moreover, the final field of view for in situ imaging is typically small.^[^ ^6^ ^]^ Therefore, they are not suitable for the rapid analysis of large‐scale biological samples.

A critical challenge of in situ imaging is the restricted movement of nucleic acids during enzymatic amplification. Cells are highly crowded structures that undergo diverse complex physiological processes.^[^ ^4^, ^7^ ^]^ The crowding and nanoconfined spaces inside the cells ensured that each biological reaction could occur in situ at their respective spatial locations without interference. As soft materials, hydrogels contain cross‐linked networks with large amounts of water,^[^ ^8^ ^]^ its 3D porous structure is similar to that of a cellular matrix. The biomimetic environment of the hydrogel allows rapid enzymatic reactions,^[^ ^9^ ^]^ whereas its nanoporous structure restricts nucleic acid diffusion.^[^ ^10^ ^]^ Meanwhile, the rotatable polymer chains in the hydrogel ensure flexible properties that fit well with rough samples.

**Figure 1:**
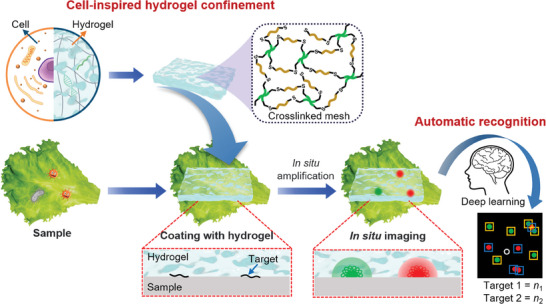
Schematic illustration of hydrogel iSCIA for in situ imaging of nucleic acids on biological samples.

Inspired by the cellular matrix, this study achieved the in situ amplification and imaging of single nucleic acid on diverse biological samples using a piece of hydrogel, as illustrated in **Figure** **1**. The entire process for in situ imaging was simple (one step) and fast (<20 min), without formaldehyde fixation. The cross‐linked polyethylene glycol (PEG) hydrogel was flexible and fitted well with a rough sample. Because of the introduction of hydrogel meshes, the target nucleic acids on the diverse biological samples were immobilized at their initial location, whereas enzymatic amplification could still be successfully performed, making this approach ideally suitable for in situ amplification and imaging. Restricted diffusion of nucleic acids was observed in the confined spaces of nanoporous hydrogels. With in situ space‐confined interfacial amplification (iSCIA), the spatial location of the target can be lighted‐up and observed within 20 min, with a detection limit as low as 1 copy/10 cm^2^. In situ multiplex imaging with a deep learning model has also been established to automatically analyze multiple targets. Finally, the developed iSCIA was successfully applied for in situ imaging of viruses, bacteria, and fungi on plant leaves and foods to realize plant health monitoring and food safety surveillance.

## 2 Results and Discussion

### 2.1 Characterization and Imaging of Hydrogels

**Figure 2:**
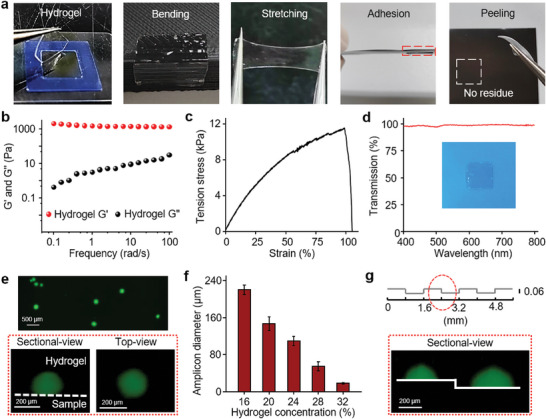
Characterization and imaging of flexible hydrogels. a) Photographs of a flexible hydrogel in bending, stretching, adhesion, and peeling on solid samples. b–d) Dynamic frequency sweep curves (b), tensile stress–strain curves (c), and transmission spectra (d) of PEG hydrogels. e) Cross sectional‐view and top‐view LSCM images of amplicons on the microscope slide. f) Amplicon diameters with different hydrogel concentrations (*n* = 3). Error bars represent the standard deviation from three independent experiments. g) Scheme of rough PDMS mould. Cross sectional‐view LSCM image of amplicons on uneven PDMS mould.

PEG hydrogels containing *Bst* polymerase, primers, and fluorescent dyes were prepared at room temperature for 2 min through Michael addition between the two PEG monomers (Figure S1, Supporting Information). As shown in **Figure** **2**a, the prepared hydrogels showed excellent flexibility (e.g., bending, stretching, adhesion, and no residue after peeling), fit well, and formed intimate conformal contact on various solid samples with rough surfaces. The storage modulus (G′) of hydrogel was considerably greater than the loss modulus (G″), indicating the high elasticity and the low fluidity (Figure 2b). The good stretchability (99.38%) was attributed to covalent cross‐linking between 4 Arm‐PEG‐acrylamide and SH‐PEG‐SH (Figure 2c). Moreover, the PEG hydrogels were highly transparent without light scattering or adsorption, which was beneficial for in situ optical sensing (Figure 2d). A more detailed characterization of the PEG hydrogels is presented in Figure S2 (Supporting Information). These excellent mechanical properties of hydrogels fulfill the requirements for the intimate coating of diverse samples in practical applications.

The entire process for in situ imaging of nucleic acid on the biological samples was simple (one step) and fast (< 20 min) without the requirement of sample pretreatment (e.g., paraformaldehyde fixation), as illustrated in Figure 1. A flexible hydrogel with biological reagents inside (e.g., *Bst* polymerase, primers, dNTP, and SYBR Green dye) was coated onto the sample and incubated at a constant temperature for 10−20 min to perform in situ loop‐mediated isothermal amplification (LAMP). For single‐bacterial imaging, lysozyme was included in the hydrogels for effective cell wall lysis.^[^ ^11^ ^]^ While for viral imaging, a reverse transcriptase was used for RT‐LAMP.^[^ ^12^ ^]^ With the introduction of crosslinked hydrogel meshes, target nucleic acids on the sample were immobilized at their initial location without further movement, whereas biological reagents (dNTPs, primers, etc.) could diffuse freely to trigger the enzymatic amplification reaction. After amplification, numerous amplicons were produced in situ and confined using nanoporous meshes, forming a series of bright fluorescent dots at the original positions for imaging.

The iSCIA was first demonstrated by spatial imaging of SARS‐CoV‐2 on a prepared glass slide (Figure 2e). Before the iSCIA, SARS‐CoV‐2 was randomly placed on glass slides. After iSCIA, a hemispheroid‐shaped amplicon was formed at the interface between the hydrogel and sample surface, indicating successful interfacial amplification (Figure 2e). The number of fluorescent dots represents the number of nucleic acids on the sample, and the position of the fluorescent dots indicates the original position of the nucleic acids. As the concentration of the target nucleic acid increased, the number of fluorescent dots gradually increased, showing a high imaging efficiency of ≈92% (Figure S3, Supporting Information). Various parameters of the PEG hydrogels were optimized to obtain the best in situ imaging results (Figure S4, Supporting Information). The sizes of fluorescence dots could be controlled easily from 15 to 300 µm by changing hydrogel nanopore sizes (Figure 2f) or amplification time (Figure S5, Supporting Information). Smaller dots could be applied for high‐resolution imaging of small‐sized samples, whereas large‐sized amplicon dots were more suitable for rapid in‐field tests directly on a large‐scale sample using smartphones or the naked eye. Indeed, a single SARS‐CoV‐2 virus on a large‐scale surface (10 cm^2^) was imaged within 20 min using the developed iSCIA, with a detection limit downed to 1 copy/10 cm^2^ (Figure S6, Supporting Information). Interestingly, these amplicons showed an evident boundary between two adjacent points, suggesting the perfect imaging of a nucleic acid (Figure S7, Supporting Information).

Owing to its flexible and soft properties, hydrogels can form intimate conformal contacts on various rough substrates. In situ imaging on a nonplanar substrate was demonstrated using a rough polydimethylsiloxane (PDMS) surface with groove arrays that were 60 µm high (see Figure S8, Supporting Information for details). As shown in Figure 2g, the SARS‐CoV‐2 RNA on a rough surface could also be in situ imaged at different heights. Furthermore, for porous samples, such as the biological tissues and porous membranes, internal nucleic acids could also be successfully imaged (Figure S9, Supporting Information). In this case, the hydrogel sol‐gel precursors were coated onto the sample, allowing penetration and infiltration. Nucleic acids inside the tissues and porous membranes could be imaged. All these results demonstrate that hydrogel iSCIA is a rapid, sensitive, and flexible technique that can be applied for in situ rapid imaging of single nucleic acid and single pathogen.

### 2.2 Characterization of Nucleic Acid Nanoconfined Movement in Hydrogels

**Figure 3:**
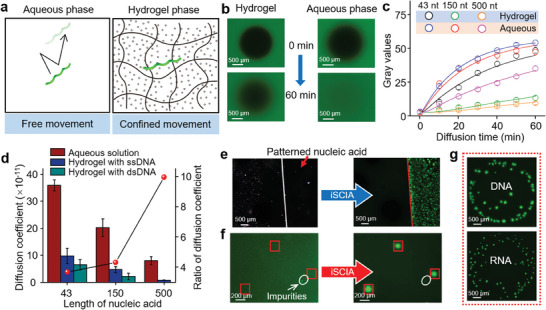
Nanoconfined movement of nucleic acids in hydrogels. a) Schematic diagram of nucleic acid movement in aqueous phase and hydrogel phase. b) Fluorescence images of nucleic acid in hydrogel and aqueous solution after photobleaching for 0 and 60 min. c) Fluorescence recovery kinetic curves of nucleic acids of 43, 150, and 500 nt in hydrogel and aqueous solution (*n* = 3). The diffusion dynamics curves were fitted by the actual fluorescence results. Error bars represent the standard deviation from three independent experiments. d) Diffusion coefficient of nucleic acids with different lengths (*n* = 3). Error bars represent the standard deviation from three independent experiments. e) Bright‐field (left) image of patterned nucleic acid before hydrogel iSCIA. Fluorescence image (right) of patterned nucleic acid after hydrogel iSCIA. f) Fluorescence images of single stained bacteria before iSCIA (Left), and single amplicon after iSCIA (Right). g) Fluorescence images of DNA and RNA “coffee ring” in drying single droplet after iSCIA.

In situ imaging of target nucleic acids at their original positions is a considerable challenge, because of the high mobility of nucleic acids in solution‐based assays (**Figure** **3**a), which easily results in the loss of the original spatial information. Although paraformaldehyde has been widely used to fix nucleic acids, it takes days for all pretreatments to occur.^[^ ^10^, ^13^ ^]^ Subsequent amplification was greatly suppressed by paraformaldehyde fixation.^[^ ^5a,c^ ^]^ The restricted movement of targets within the nanoporous hydrogel matrix is the foundation of in situ imaging.^[^ ^14^ ^]^ The prepared crosslinked hydrogel is a nanoporous material with a pore size of ≈20 nm (Figure S10, Supporting Information). Because nucleic acids are long‐chain molecules with highly complex 3D single‐stranded or double‐stranded structures, their diffusion in the cross‐linked hydrogel network was remarkably suppressed (Figure 3a). The nanoconfined movement behavior of the nucleic acids inside the nanoporous hydrogel was characterized by photobleaching experiments (see Experimental Section for details).^[^ ^11^ ^]^ Briefly, the hydrogel loaded with fluorescently labeled nucleic acids was exposed to blue light for 10 min, resulting in a patterned region of weak fluorescence (Figure 3b). Diffusion of nucleic acids induces fluorescence recovery over time. As shown in Figure 3b, the bleached pattern in the aqueous solution recovered gradually, whereas that in the hydrogel remained unchanged, demonstrating the restricted movement of nucleic acids inside the crosslinked hydrogel networks. In fact, the restriction factor correlated with nucleotide length. To quantify the diffusion coefficient (*D*) of nucleic acids in the nanoconfined space, fluorescence recovery kinetic curves were plotted (Figure 3c; Figures S11 and S12, Supporting Information). The final parameter *D* was obtained by fitting the curves (see Note S1, Supporting Information for details). As shown in Figure 3d, the diffusion coefficient *D* of the nucleic acid inside the hydrogel (*D*_h_) was lower than that in the aqueous solution (*D*_w_). The restriction factor, defined as *D*_h_/*D*_w_, increased with longer nucleic acid lengths, implying a strong nanoconfined interaction between the cross‐linking hydrogel networks and long‐chain nucleotides. With a smaller pore size, the PEG hydrogel imposed a more significant restriction on nucleic acid movement, and smaller dots were observed (Figure S12, Supporting Information). Compared to single‐stranded nucleic acids, double‐stranded DNA showed a larger restriction factor, which might be due to the straight long chain structures (Figure 3d). Restricted diffusion in the hydrogel suggests the possibility for in situ amplification.

To demonstrate that hydrogels iSCIA could reveal the exact location of nucleic acids, we explored the spatial position of the targets before and after iSCIA. Nucleic acids pre‐patterned on the substrates were successfully imaged at the same position with similar pattern shapes (Figure 3e; Figure S13, Supporting Information). To further investigate the spatial imaging capacity, the location of a single bacterium was imaged before and after the hydrogel iSCIA. Without amplification, single bacteria stained with SYBR Green were observed as a faint fluorescent signal using a high‐resolution fluorescence microscope (Figure 3f, left). After iSCIA, the amplicon could be clearly recognized and located at the same position as before (Figure 3f, right), without paraformaldehyde fixation or other sample pretreatments. The fluorescence signals were so strong that they could be easily distinguished from background impurities (Figure 3f, white circle) and observed directly by the naked eye (Figure S14, Supporting Information). In situ imaging ability of hydrogel iSCIA was then demonstrated by imaging nucleic acid “coffee‐ring.” As shown in Figure 3g, after droplet drying, nucleic acids were distributed at the edge rather than inside of loop, in analogy with the colloidal “coffee‐ring.”^[^ ^15^ ^]^ These experiments clearly illustrate that the hydrogel iSCIA is capable of correctly imaging the physical location of targets.

### 2.3 Multiplex Imaging with Deep Learning for Automated Discrimination

**Figure 4:**
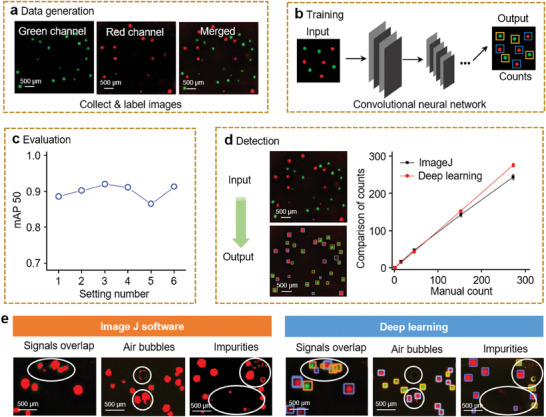
Multiplex imaging with deep learning for automated discrimination. a) Collected fluorescence images with green channel, red channel, and merged after multiplex hydrogel iSCIA. Green channel and red channel were in situ imaging of *Escherichia coli* and *Listeria monocytogenes*, respectively. b) The model training by a convolutional neural network (CNN) for classification and prediction. c) Model evaluation via mean average precision 50 (mAP 50). d) Model detection for new images (left). Comparation of counts using deep learning, image J software, and manual count (right) (*n* = 3). Error bars represent the standard deviation from three independent experiments. e) Processed fluorescence images with multi‐signals overlap, air bubbles, and impurities by image J software and deep learning.

To achieve multiplex in situ imaging of different targets using the hydrogel iSCIA, the quenching of unincorporated amplification signal reporters (QUASR) was applied.^[^ ^16^ ^]^ The fluorescence‐labeled forward inner primers (FIPs) were integrated into the DNA amplicons as the template expanded, whereas the free FIPs were quenched by the quencher‐labeled probes. Multicolor channels paired with multiple molecular beacons were obtained and merged from one piece of hydrogel. Excellent specificity and selectivity were confirmed using a multiplex imaging system (Figure S15, Supporting Information). As shown in **Figure** **4**a, the spatial location of multiple pathogens (*Escherichia coli* and *Listeria monocytogenes*) could be exhibited simultaneously using the hydrogel iSCIA.

To accurately discriminate the target signals while avoiding interference, artificial intelligence‐based image processing was conducted. Artificial intelligence is a powerful tool for accurately discriminating positive signals.^[^ ^2^, ^17^ ^]^ We trained the deep learning (DL)‐assisted YOLOv8m model for the auto‐processing of multiplex images (Figure 4a–d). More detailed studies on the optimization and evaluation of the hyperparameters and configurations are presented in Figures S16–S19 and Tables S1 and S2 (Supporting Information). With the optimal model configuration, the mean average precision (mAP) could reach 0.92 (Figure 4c), suggesting good model performance in precision and recall. As shown in Figure 4e, multi‐signal dots that overlapped with high spatial proximity were precisely identified using the multiplex DL model, and occasional bubbles or other fluorescent impurities were automatically excluded (Figure 4e, white circle). In contrast, amplicon dots close to each other cannot be distinguished by conventional imaging processing (e.g., ImageJ), and air bubbles or other interference signals can also cause false‐positive and false‐negative results. As a result, ImageJ discrimination was mostly inaccurate (R^2^ = 0.9896), whereas our DL model showed high accuracy for automated discrimination (R^2^ = 0.9999), with a rapid recognition speed of 2−5 s for each image (Figure 4d).

### 2.4 Performance of Hydrogel iSCIA on Diverse Substrates

**Figure 5:**
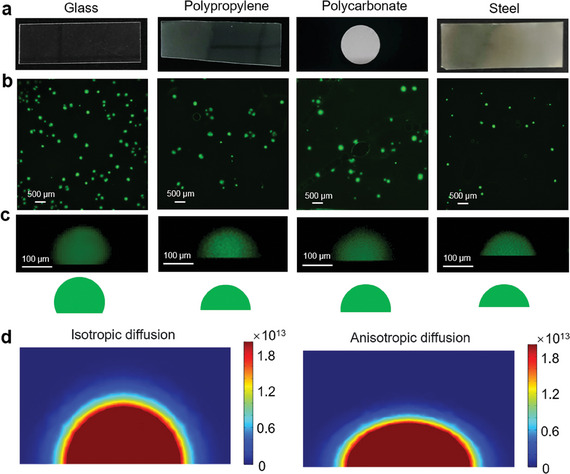
In situ imaging on diverse materials. a) The photograph of glass, polypropylene, polycarbonate, and steel. b,c) Top‐view (b) and cross sectional‐view (c) fluorescence images of amplicons on glass, polypropylene, polycarbonate, and steel after iSCIA. d) COMSOL simulation of amplicons in iSCIA with isotropic diffusion coefficient (left) and anisotropic diffusion coefficient (right).

The developed iSCIA was successfully applied for in situ imaging of SARS‐CoV‐2, with single‐virus sensitivity on various materials, including glass, polymers (PDMS, polypropylene, polycarbonate, and polyvinyl pyrrolidone), and metals (Au, Al, and steel) (**Figure** **5**a,b; Figure S20, Supporting Information). Good reproducibility of the iSCIA system across different samples was demonstrated (Figure S21, Supporting Information). Interestingly, the interfacial morphologies of the amplicons on the different materials possessed different shapes, as shown in the cross‐sectional images (Figure 5c; Figure S20, Supporting Information). For example, the dots generated on polypropylene or polycarbonate were hemispherical, whereas a semi‐ellipse was observed on aluminum and steel. This is because the morphology of the dots depends on two processes: nanoconfined amplification and restricted diffusion of nucleic acids. Because the affinity between nucleic acids and various substrates is different, the diffusion coefficients of nucleic acids in the longitudinal (↑) and transversal (→) directions are discrepant. We conducted a finite element analysis using COMSOL to simulate the morphology of amplicon dots under interfacial nanoconfined amplification with an anisotropic diffusion coefficient. As shown in Figure 5d, with an increase in nucleic acid affinity, the morphology of the dots changed from hemispherical to semi‐elliptical.

The developed iSCIA system showed the high stability at various temperatures and pH levels (Figure S22, Supporting Information). After storage of the flexible hydrogel system at 4 °C for 1 month, in situ imaging was still successful, indicating its long‐term stability and potential for practical in situ imaging (Figure S23, Supporting Information). The hydrogel iSCIA could also be successfully applied for the rapid imaging of SARS‐CoV‐2 virus contamination on diverse transmission media samples, including packages, food containers and express (Figure S24, Supporting Information). This will be of beneficial for tracing the source of pathogens and potential virus carriers contaminating environmental media. Traditional methods require swabbing steps to collect targets from the surface into an aqueous solution, which is labor‐intensive, time‐consuming, and lacks location information. The developed iSCIA achieved in situ imaging of pathogens directly on the samples in 20 min without tedious pretreatment.

### 2.5 In Situ Imaging of Pathogens on Biological Samples

**Figure 6:**
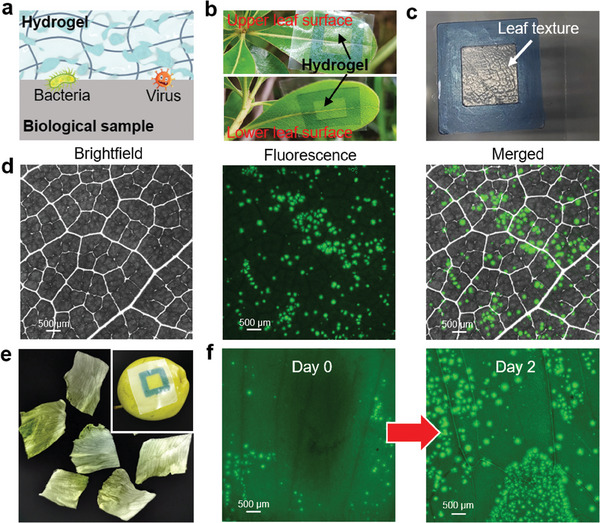
In situ imaging of pathogens on plant, vegetable, and fruit samples. a) Schematic diagram of in situ confinement of pathogens by coating hydrogel on biological samples. b) Photograph of *Pittosporum tobira* leaf coating with flexible hydrogel. c) Photograph of flexible hydrogel detached from leaf. d) Bright‐field, fluorescence, and merged images of hydrogel iSCIA for imaging *Alternaria alternata* directly on *Pittosporum tobira*. e) Photograph of lettuce and pear. f) Fluorescence iSCIA images of *E. coli* on uneven lettuce with infection time.

Rapid in situ imaging of pathogens on foods, plants, and other biological samples is conducive to ensuring plant health and food safety.^[^ ^18^ ^]^ The hydrogel system was first used to monitor plant diseases in‐field (**Figure** **6**a,b). The pathogens on the leaf surface were in situ amplified by iSCIA. These DNA products were stained with SYBR Green dye, which was pre‐embedded in the hydrogel. The fluorescence signals produced by the iSCIA were so strong that they could be distinguished from the leaf fluorescence background without any pretreatment. After coating plant leaves (*Pittosporum tobira*), the flexible hydrogel showed an identical leaf texture, indicating intimate conformal contact between the hydrogel and the biological samples (Figure 6c). After *Alternaria alternata* infection, fluorescent dots were clearly visible on the leaves of *P. tobira* with merged bright‐field images showing the spatial distribution of pathogens (Figure 6d). Pathogens were more likely to be located on the foliar surface. For plant health monitoring, hydrogels could also be peeled off from plant leaves for imaging. In this case, the self‐fluorescent background of the biological samples could be eliminated, and plant health could be monitored using the attached hydrogel (Figure S25, Supporting Information).

In addition to monitoring plant health, in situ imaging has been applied directly to foods carrying foodborne pathogens. Freshly cut lettuce is easily infected with *E. coli*. As shown in Figure 6e,f, the presence of *E. coli* on lettuce was observed with spatial distribution information within 20 min using a piece of hydrogel without complex sample pretreatments. The merged fluorescence and bright field images are shown in Figure S26 (Supporting Information). At an early stage, the number of *E. coli* cells in lettuce was low and only located on the periphery near the cutting wound. Over time, the number of pathogens grew rapidly and spread to infect the whole lettuce (Figure 6f). Similar results were obtained for Green Tea and fruits with pathogen infection (Figures S27 and S28, Supporting Information). After attaching the hydrogel to the surface of the fruits, the presence of SARS‐CoV‐2 was directly imaged on the cold‐chain fruits, including pears, cherries, longans, pitaya, and bananas (Figure 6e; Figure S28, Supporting Information). Furthermore, we investigated the performance of the iSCIA system for imaging pathogens from actual environmental samples with naturally occurring pathogens, such as the norovirus GI in ground soil water and *E. coli* contamination on plastic wrap that covers fresh food in retail supermarkets (Figure S29, Supporting Information).

Compared with existing in situ imaging methods, our iSCIA technique has many advantages in terms of sample pretreatment, detection time, imaging efficiency, cost, and ease of use (Table S3, Supporting Information). First, the spatial distribution of single molecule and single pathogen on real solid‐state samples can be in situ imaged <30 min without sample pretreatment (e.g., pre‐fixation). While conventional in situ imaging techniques require sample pre‐fixation and at least 10 h for all detection procedures. Second, all steps, including cell lysis, nucleic acid fixation, amplification, and imaging, were integrated into a piece of hydrogel, which substantial simplified the entire procedure and enhanced imaging efficiency. In addition, the solid‐state samples were detected directly by attaching a hydrogel without the need for a swabbing step, indicating the ease of use for point‐of‐care in‐field detection. Furthermore, its low cost ($0.85) and high universality offer the possibility of diverse targets imaging, such as nucleic acids, bacteria, fungi, and viruses, on different biological samples.

## 3 Conclusion

In this study, we present a flexible PEG hydrogel for in situ imaging of pathogens on diverse solid‐state biological samples. The flexible PEG hydrogel exhibited intimate conformal contact with the rough samples. By coating the hydrogel onto the sample, the movement of nucleic acids inside the nanoporous hydrogel was strongly confined, and in situ interfacial amplification was successfully achieved in 20 min with a detection limit of 1 copy/10 cm^2^. The nanoconfined diffusion behaviors of different nucleic acids in the nanoporous hydrogel were characterized in detail. Combined with deep learning, fluorescent hydrogel images could be transduced into data for accurate discrimination and multiplex in situ imaging. Benefiting from the powerful in situ‐confined enzymatic reaction, hydrogel iSCIA could be applied to diverse chemical substrates with high universality. By attaching a hydrogel on plant leaves, plant health was successfully monitored and pathogens on the leaves were in situ imaged. Moreover, the presence of *E. coli* on lettuce was in situ observed, showing time and spatial‐resolved infection of pathogens. The proposed technique, as a flexible monitoring sensor for in situ imaging of nucleic acids, bacteria, and viruses, is very promising for plant health monitoring, food safety detection, and environmental surveillance.

We believe that this simple and rapid in situ imaging method offers many potential opportunities for non‐specialized individuals without long‐term training. Additional applications of pathogens screening will be inspired by various scenarios and fundamental research. The simultaneous detection of multiple components can be achieved using a multiple‐chamber array without cross‐contamination. Furthermore, iSCIA can be paired with stimuli‐responsive matrices to achieve novel functions, such as in situ sequencing, molecular exchange, or spatial transcriptomics. This iSCIA technique can serve as an ideal and simple platform for in situ monitoring and screening of any target directly on samples, which is of great significance for health development in biomedicine, agriculture, sanitation, the environment, and the food industry.

## 4 Experimental Section

### Chemicals and Materials

All LAMP and RT‐LAMP reagents were purchased from New England Biolabs (Ipswich, MA, USA). The sequences of primers, probes, and SARS‐CoV‐2 RNA were obtained from Sangon Biotechnology (Shanghai, China). SARS‐CoV‐2 virus was purchased from Xiamen Zeesan Biotech (Xiamen, China). All the bacterial strains were obtained from the China Center of Industrial Culture Collection (CICC, Beijing, China). The fluorescent dye (SYBR Green I) for nucleic acid staining was purchased from Thermo Fisher Scientific (San Jose, CA, USA). Frame seals were obtained from Bio‐Rad (Hercules, California, USA). All hydrogel monomers, like 4‐Arm PEG acrylate (average molecular weight (AMW) 10000) and SH‐PEG‐SH (AMW 3400), were purchased from Laysan Bio (Arab, Alabama), stored as lyophilized powders, and dissolved in sterile water for utilization.

### Cell Culture

*Listeria monocytogenes* CICC 21633 and *Escherichia coli* CICC 10907 were preserved with glycerol at −80 °C. They were reactivated with Brain Heart Infusion broth and Luria‐Bertani broth, respectively, in the shaking incubator at 37 °C for overnight. The cultured bacteria were centrifuged at 12 000 rpm for 3 min, and resuspended in sterile deionized water for later use.

### In Situ Space‐Confined Interfacial Amplification Protocol

To prepare the iSCIA assay, 1.6 mg 4 Arm‐PEG‐acrylate, 1.1 mg SH‐PEG‐SH, 1 × isothermal buffer, 1.6 µM FIP and BIP, 0.2 µm F3 and B3, 0.4 µm LF and LB, 1.4 mm dNTP, 6 mm total MgSO_4_, 16 U *Bst* 2.0 WarmStart polymerase, and 1 × SYBR Green I were mixed and introduced into a frame‐seal chamber for cross‐linking and gelation at room temperature. For bacteria analysis, 0.1 mg mL^−1^ lysozyme was included in LAMP system for cell wall lysis.^[^ ^11^ ^]^ For virus analysis, the RT‐LAMP assay was conducted by adding extra 7.5 U of reverse transcriptase to the assay mentioned above. The primer sets used to amplify the SARS‐CoV‐2, *A. alternata, E. coli*, and *L. monocytogenes* are listed in Table S4 (Supporting Information). SARS‐CoV‐2 randomly located on a glass slide was prepared as a typical solid‐state sample for in situ imaging.

To conduct iSCIA, the prepared hydrogel containing all reagents was coated onto the samples in sealed chambers, followed by incubation at 65 °C for 10−20 min to conduct isothermal amplification. After amplification, the fluorescent SYBR Green dye was bound to the nucleic acid products, generating fluorescence signals. Thus, a series of bright fluorescent points were observed at their original locations under blue light.

### Photobleaching Test

Hydrogels loaded with fluorophore‐labeled nucleic acids were exposed to blue light for 10 min, resulting in a circular region with weak fluorescence. Over time, the fluorescence of the bleached pattern gradually recovered, and images were captured and recorded using a fluorescence microscope. The diffusion processes of nucleic acids of different lengths were explored using fluorescence recovery curves after photobleaching. The diffusion rate of nucleic acids and the diffusion coefficient were calculated by comparing the degree of fluorescence recovery for various nucleotide lengths. Three experiments were performed at different positions during the photobleaching tests.

### Single‐Bacteria Imaging before and after Hydrogel iSCIA

To confirm the preservation of spatial information of targets after hydrogel iSCIA, single bacteria were imaged before and after the iSCIA assay. A bacterial sample was first stained with 1 × SYBR Green I for 30 min, its location on a microscope slide was recorded, and iSCIA was performed. The position of a single bacterial cell was observed and compared to that of a single amplicon after iSCIA.

### Multiplex Imaging Assay

The QUASR was introduced and optimized in a multiplex imaging assay.^[^ ^16b^ ^]^ The 5′‐end FIPs of *E. coli* was labeled with the FAM, while the quenching probe was designed by a complementary sequence versus FIP, with the TAMRA at the 3′‐end. Similarly, the 5′‐end FIPs of *L. monocytogenes* was labeled with the ROX, while the quenching probe was designed by a complementary sequence and modified with the BHQ2 at the 3′‐end. The probe concentration was optimized to obtain the highest signal‐to‐noise ratio. End‐point hydrogel images were captured under the green and red channels and then merged into a picture using ImageJ (NIH, MD) software for subsequent analysis.

### Deep Learning‐Assisted Image Analysis

For automated amplicon discrimination, the YOLOv8m algorithm and convolutional neural network (CNN) were used to establish the DL model. First, 22 single‐color fluorescence images were manually annotated using the RoboFlow platform. The dataset of these images was randomly divided into a training set and a validation set in a 10:1 ratio. Six sets of hyperparameters and configurations were optimized and evaluated. Then, a new image was analyzed using the DL model to assess the accuracy of the model‐recognized signals, which were compared with the ImageJ software and manual counts. In addition, ten multiplex fluorescent images were used to train the DL model to discriminate the multiplex in situ images, and the diverse signals in the green and red channels were labeled.

### Plant Health Monitoring and Food Safety Surveillance

Plant diseases caused by pathogens were diagnosed using the developed iSCIA. *Pittosporum tobira* leaves were randomly sampled from the campus of Zhejiang University. The prepared hydrogel was coated directly onto plant leaves. After isothermal incubation, amplicon dots were generated, which indicated the location of the pathogens on the plants. Detailed information regarding the peel‐off of the iSCIA is presented in Figure S25 (Supporting Information).

Vegetables (lettuce) and fruits (pears, cherries, longans, pitaya, and bananas) were purchased from a local supermarket. The iSCIA system was prepared by mixing the hydrogel monomers and isothermal reaction reagents as discussed above, which coated on these biological samples, and incubated at 65 °C for 15–20 min. Subsequently, the spatial distribution of pathogens on these biological samples was in situ observed.

### Characterization

SEM image of hydrogel mesh was obtained using a field‐emission scanning electron microscope (S‐4800, Hitachi High‐Technologies, Tokyo, Japan). The elasticity and storage modulus of the hydrogels were measured using a rheometer (DC‐2006A, Anton Paar Trading, Shanghai, China). Fluorescent images were obtained using an inverted fluorescence microscope (DMi8, Leica Biosystems, Germany). The top‐view and cross‐sectional amplicon images were captured using a laser confocal fluorescence microscope (Leica TCS SP8, Leica Biosystems, Germany). The UV–vis spectra were obtained using a microplate reader (200 PRO, Tecan, Grodig, Austria). The tensile stress‐strain curve was measured with 10 mm min^−1^ using a universal material‐testing machine (Z020, ZwickRoell, Germany). Hydrogels on various materials were stretched to separate at 50 mm min^−1^ to obtain the adhesion strength.

### COMSOL Simulation

Finite element modeling was performed using the commercial COMSOL Multiphysics 5.2 (COMSOL Inc., Burlington, MA). In the simulations, the interfacial morphologies of the amplicon dots on different substrates were represented using a 2D model. Two procedures, the amplification and diffusion of nucleic acids, were considered inside the hydrogel with different diffusion coefficients, as calculated from the photobleaching tests. Confined spaces in hydrogel for nucleic acid movement were represented using the 500 × 500 × 500 µm scale. The constant of amplification was set as 2 min^−1^ to represent the amount of nucleic acid replication. The diffusion source for the nucleic acid template was a particle with a radius of 100 nm. Its initial concentration was set as 0.01 mm, which was similar to 0 mm compared to that of amplified products. The behavior of the nucleic acid particles was calculated, and the amplicon shape on the substrate was exhibited.

### Statistical Analysis

Statistical analyses were performed using Microsoft Excel 2019 (Microsoft Corporation, Redmond, Washington, USA). All data were represented as the mean ± standard deviation (SD), unless noted otherwise. No statistical methods were used to pre‐determine the sample size, which was annotated in the context or figure captions. For two‐group comparisons, statistical significance was determined using a two‐tailed unpaired Student's t‐test. Statistical significance was set at *p* < 0.05. Statistically significant results were indicated in the figures using *p < 0.05, **p < 0.01, ***p < 0.001, ****p < 0.0001, and no significance (n.s., *p* > 0.05).

## Conflict of Interest

The authors declare no conflict of interest.

## Supporting information



Supporting Information

## Data Availability

The data that support the findings of this study are available from the corresponding author upon reasonable request.
