# Supplementary material for: Space‐Confined Amplification for In Situ Imaging of Single Nucleic Acid and Single Pathogen on Biological Samples
Source: Adv Sci (Weinh). 2024 Oct 7;11(44):2407055. doi: 10.1002/advs.202407055 (PMC11600185; doi:10.1002/advs.202407055)
Supplement: Supplementary file 1 — Supporting Information [file ADVS-11-2407055-s001.docx]

Supplementary Information

**Space-Confined Amplification for *In Situ* Imaging of Single Nucleic Acid and Single Pathogen on Biological Samples**

*Tao Yang, Dong Li, Zisheng Luo, Jingjing Wang, Fangbin Xiao, Yanqun Xu, Xingyu Lin^*^*

**Table of Contents**

**Note S1** 3

**1 Gelation analysis** 10

1.1 Principle and process of gelation 10

1.2 Thermal stability and adhesive strength of hydrogel 10

**2 Hydrogel iSCIA analysis** 12

2.1 Imaging efficiency 12

2.2 *In situ* imaging with high concentration of hydrogel and different time 13

2.3 Single-molecule imaging on a large-scale sample 13

2.4 Boundary of two adjacent amplicon points 14

2.5 Fabrication of PDMS with rough surface 14

2.6 *In situ* imaging on porous membrane and mouse brain tissue 15

2.7 Scanning electron microscope image of hydrogel 15

**3 Confined movement of nucleic acids in hydrogel** 17

3.1 Photobleaching experiment 17

3.2 Comparison of diffusion process 18

3.3 Patterned molecular imaging 18

3.4 Naked eye-based observation 18

3.5 Specificity evaluation 19

**4 Deep learning for automatically counting** 21

4.1 Dataset generation 21

4.2 Performance evaluation 21

4.3 Comparison of counting process 24

**5 Performance of hydrogel iSCIA** 25

5.1 Cross-sectional shape of amplicon on various materials 25

5.2 Reproducibility across different samples 25

5.3 Stability of iSCIA in different conditions 26

5.4 Long-term stability after storage and transportation 26

5.5 *In situ* imaging of pathogens on diverse real samples 27

**6 *In situ* imaging on biological samples** 28

6.1 Hydrogel peeling-off iSCIA 28

6.2 *In situ* imaging of *E. coli* O157:H7 infection on lettuce 28

6.3 *In situ* imaging of *E. coli* on Green Tea 29

6.4 *In situ* imaging of SARS-CoV-2 on cold-chain fruits 29

6.5 Practical detection from environmental samples 29

**7 Sequences of primers** 31

**References** 33

**Note S1**

The detailed derivation and calculation of the diffusion equation for nucleic acid were presented in the finite concentration trap model, which included the establishment of equation and the solution of equation with definite condition.

**Establishment of equation**

**Fick's second law**

When the following model is considered, the entire plane is filled with a solvent with a diffusion coefficient of *D* for DNA. The initial distribution of DNA concentration is 0 in the circular region of radius *ρ_0_*, and the uniform distribution of concentration is *c_0_* outside this region. The distribution of concentration field *c* in the plane at time t is investigated.


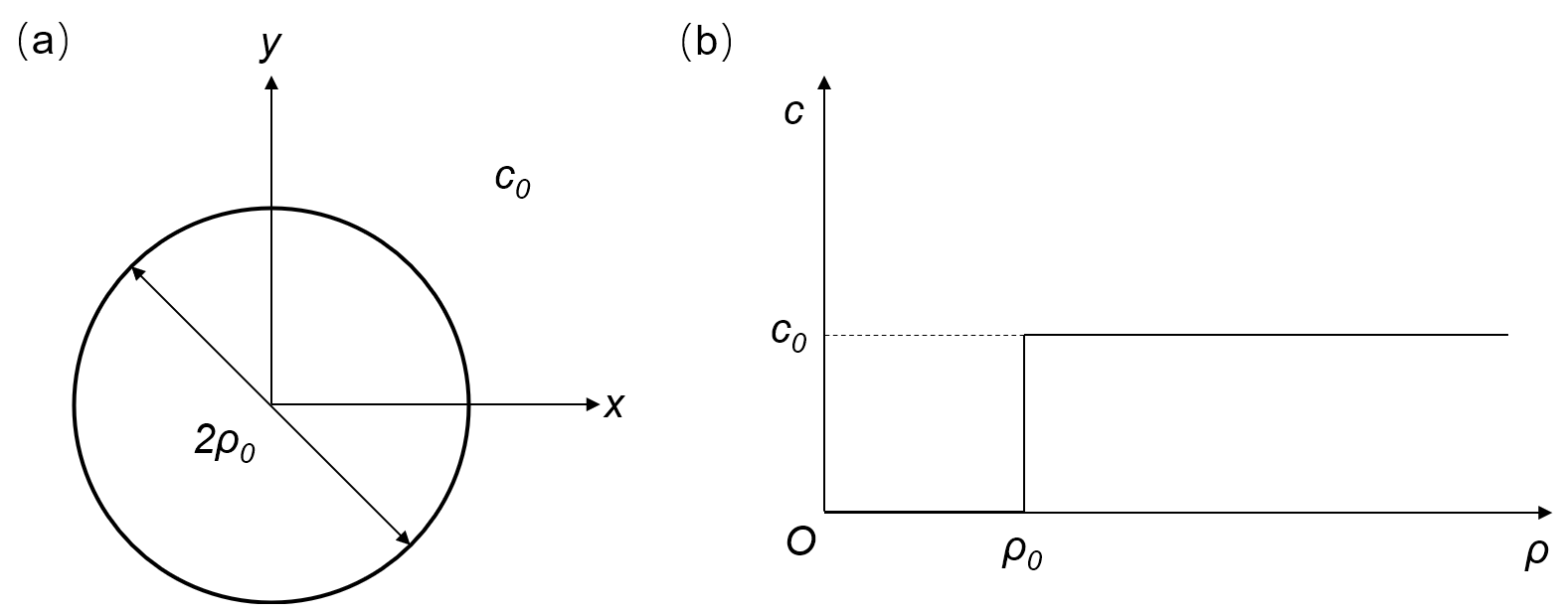


**Figure note** **S1.1.** (a) Finite concentration trap model (b) Distribution of the distance between the concentration pairs and the center at the initial time

The solution of the concentration distribution under this model involves the diffusion equation, which is Fick's second law:

$$\begin{aligned} \frac{\partial c}{\partial t}-D\Delta c=F\#\left（ SI.1 \right） \end{aligned}$$

where *F* is the strength of the source in the diffusion field. Since there is no source of diffusion in the case, we take $F=0$ here. The diffusion equation can be simplified as

$$\begin{aligned} \frac{\partial c}{\partial t}-D\Delta c=0\#\left（ SI.2 \right） \end{aligned}$$

**Transformation of coordinate system**

In the planar diffusion, the two-dimensional diffusion equation can be defined as^[1]^

$$\begin{aligned} \frac{\partial c}{\partial t}=D\left( \frac{\partial^{2}c}{{\partial x}^{2}}+\frac{\partial^{2}c}{{\partial y}^{2}} \right)\#\left（ SI.3 \right） \end{aligned}$$

Thereinto, the following relationship is existing.

$$\begin{aligned} x=\rho\cos\theta\#\left（ \text{SI.4a} \right） \end{aligned}$$

$$\begin{aligned} y=\rho\sin\theta\#\left（ \text{SI.4b} \right） \end{aligned}$$

$$\begin{aligned} \rho=\sqrt{x^{2}+y^{2}}\#\left（ \text{SI.4c} \right） \end{aligned}$$

$$\begin{aligned} \theta=\tan^{-1} \frac{y}{x}\#\left（ \text{SI.4d} \right） \end{aligned}$$

Their geometric relationship can be shown as Figure note S1.2.


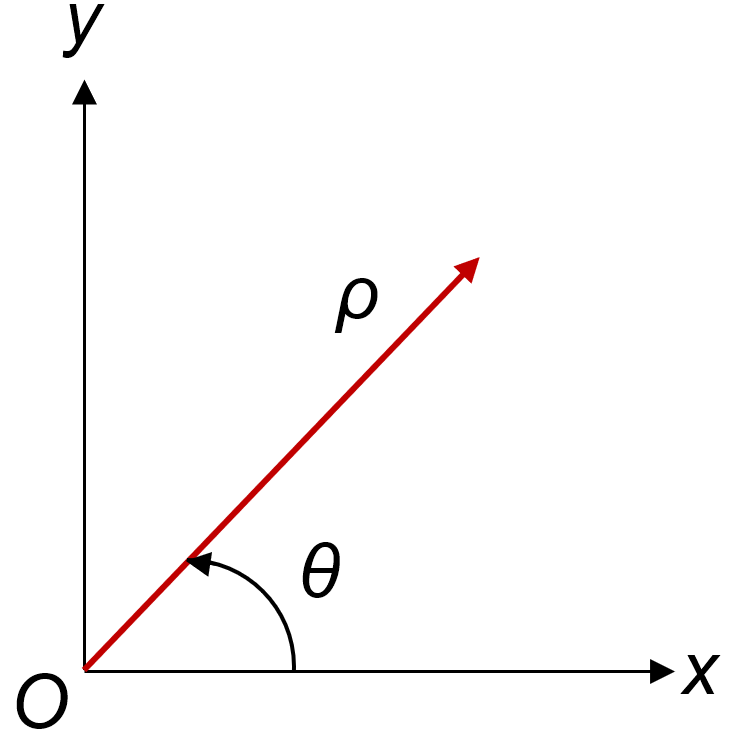


**Figure note S1.2.** Coordinate axes system for Cartesian and polar coordinates

The diffusion equation is converted from Cartesian coordinate system to polar coordinate system. So, the polar coordinate is considered as

$$\begin{aligned} \Delta=\nabla^{2}=\frac{1}{\rho}\frac{\partial}{\partial\rho}+\frac{\partial^{2}}{{\partial\rho}^{2}}+\frac{1}{\rho^{2}}\frac{\partial^{2}}{{\partial\theta}^{2}}\#\left（ SI.5 \right） \end{aligned}$$

The Equation SI.3 can be expressed as

$$\begin{aligned} \frac{\partial c}{\partial t}=D\left( \frac{1}{\rho}\frac{\partial c}{\partial\rho}+\frac{\partial^{2}c}{{\partial\rho}^{2}}+\frac{1}{\rho^{2}}\frac{\partial^{2}c}{{\partial\theta}^{2}} \right)\#\left（ SI.6 \right） \end{aligned}$$

**Separation of variable**

Since the concentration distribution is symmetric throughout the model, the following relationship is obtained.

$$\begin{aligned} \frac{\partial c}{\partial\theta}=0\#\left（ SI.7 \right） \end{aligned}$$

In this way, the Equation SI.6 can be further simplified as

$$\begin{aligned} \frac{\partial c}{\partial t}=D\left( \frac{1}{\rho}\frac{\partial c}{\partial\rho}+\frac{\partial^{2}c}{{\partial\rho}^{2}} \right)\#\left（ SI.8 \right） \end{aligned}$$

We assume that the solution has the following form

$$\begin{aligned} c\left( \rho,t \right)=R\left( \rho\right)T\left( t \right)\#\left( SI.9 \right) \end{aligned}$$

Considering the *ρ*-dependent operators act only on *R(ρ)*, the *t*-dependent operators act only on *T(t)*. By introducing Equation SI.9 into Equation SI.8, it can be expressed as

$$\begin{aligned} R\left( \rho\right)\frac{\partial T\left( t \right)}{\partial t}=D\left( \frac{1}{\rho}\frac{\partial R\left( \rho\right)}{\partial\rho}+\frac{\partial^{2}R\left( \rho\right)}{{\partial\rho}^{2}} \right)T\left( t \right)\#\left（ SI.10 \right） \end{aligned}$$

The both sides of the Equation SI.10 is divided by *R(ρ)T(t)*, and the following form is got.

$$\begin{aligned} \frac{1}{T\left( t \right)}\frac{\partial T\left( t \right)}{\partial t}=D\left( \frac{1}{\rho}\frac{\partial R\left( \rho\right)}{\partial\rho}+\frac{\partial^{2}R\left( \rho\right)}{{\partial\rho}^{2}} \right)\frac{1}{R\left( \rho\right)}\#\left（ SI.11 \right） \end{aligned}$$

The only way to maintain this equality is each side of this expression to be equal to a constant that is called as -*E*. As a result, the Equation SI.11 is separated into two equations, one dealing with time dependence (Equation SI.12a) and another with radial dependence (Equation SI.12b).

$$\begin{aligned} \frac{1}{T\left( t \right)}\frac{\partial T\left( t \right)}{\partial t}=-E\#\left（ SI.12a \right） \end{aligned}$$

$$\begin{aligned} \left( \frac{1}{\rho}\frac{\partial R\left( \rho\right)}{\partial\rho}+\frac{\partial^{2}R\left( \rho\right)}{{\partial\rho}^{2}} \right)\frac{1}{R\left( \rho\right)}=-\frac{E}{D}\#\left（ SI.12b \right） \end{aligned}$$

**Solution of the equation with definite condition**

**Solution of the time equation**

Because the time equation (Equation SI.12a) is only a function of *t*, the partial derivative is replaced by the ordinary derivative, which is expressed as

$$\begin{aligned} \frac{dT\left( t \right)}{dt}+ET\left( t \right)=0\#\left（ SI.13 \right） \end{aligned}$$

The solution to this ordinary differential equation is

$$\begin{aligned} T\left( t \right)=T_{0}e^{-Et}\#\left（ SI.14 \right） \end{aligned}$$

where $T_{0}$ is the integral constant. Here$E>0$, otherwise the solution will not converge.

**Solution of the radial equation**

Because the radial equation (Equation SI.12b) is only a function of *ρ*, partial derivative is replaced by the ordinary derivatives, which is expressed as

$$\begin{aligned} \frac{d^{2}R\left( \rho\right)}{{d\rho}^{2}}+\frac{1}{\rho}\frac{dR\left( \rho\right)}{d\rho}+\frac{E}{D}R\left( \rho\right)=0\#\left（ SI.15 \right） \end{aligned}$$

After substituting $r=\sqrt{\frac{E}{D}}\rho$,$R\left( r \right)=R\left( \sqrt{\frac{E}{D}}\rho\right)$, the derivative rule of composite function is used to obtain the following equation.

$$\begin{aligned} \frac{d^{2}R\left( r \right)}{{dr}^{2}}+\frac{1}{r}\frac{dR\left( r \right)}{dr}+R\left( r \right)=0\#\left（ SI.16a \right） \end{aligned}$$

*i.e.*

$$\begin{aligned} r^{2}\frac{d^{2}R\left( r \right)}{{dr}^{2}}+r\frac{dR\left( r \right)}{dr}+r^{2}R\left( r \right)=0\#\left（ SI.16b \right） \end{aligned}$$

This is the zero-order Bessel equation, its general solution is

$$\begin{aligned} R\left( r \right)=C_{1}J_{0}\left( r \right)+C_{2}N_{0}\left( r \right)\#\left（ SI.17 \right） \end{aligned}$$

where $J_{0}\left( r \right)$ and $N_{0}\left( r \right)$ are zero-order Bessel functions and zero-order Neumann functions, respectively. $C_{1}$ and $C_{2}$ are integral constants.

**Application of definite conditions to the solution**

According to the Figure note S1.1 (b), the definite solution condition is used to determine the constant in the solution of the equation. The concentration is finite at the center and at infinity, and the initial conditions are shown in Figure note S1.1 (b). These conditions can be expressed as follows:

1. when $\rho\to0$, $c\to finite$
2. when $\rho\to\infty$, $c\to c_{0}$
3. when $t=0$, $c=circ\left( \rho\right):=\left\{ \begin{aligned} c_{0}，\left( \rho>\rho_{0} \right) \\ 0，\left( \rho\leq\rho_{0} \right) \end{aligned} \right.$

When $r\to0$ (*i. e.* $\rho\to0$), $N_{0}\left( r \right)\to-\infty$, so $C_{2}$ is expressed as 0. That means the solution of the radial equation consists only of zero-order Bessel functions.

Since the initial condition is defined on an infinite interval, it needs to be expressed as a Fourier-Bessel integral. The form of the solution should be changed from an infinite series to an infinite integral. Then, $circ\left( \rho\right)$ is expanded into a Fourier-Bessel integral. After normalization and substituting ${circ}_{0}\left( \rho\right)=1-\frac{1}{c_{0}}circ\left( \rho_{0}\rho\right)$, the equation is obtained as following:

$$\begin{aligned} {circ}_{0}\left( \rho\right)=\left\{ \begin{aligned} 1，\left( \rho\leq1 \right) \\ 0，\left( \rho>0 \right) \end{aligned} \right.\#\left（ SI.18 \right） \end{aligned}$$

By expanding the ${circ}_{0}\left( \rho\right)$, the Fourier-Bessel integral of ${circ}_{0}\left( \rho\right)$ is expressed as

$$\begin{aligned} \left\{ \begin{aligned} {circ}_{0}\left( \rho\right)=\int_{0}^{\infty} F\left( \lambda\right)J_{0}\left( \lambda\rho\right)\lambda d\lambda\\ F\left( \lambda\right)=\int_{0}^{\infty} {circ}_{0}\left( \rho\right)J_{0}\left( \lambda\rho\right)\rho d\rho\end{aligned} \right.\#\left( \mathrm{SI}.19 \right) \end{aligned}$$

$F\left( \lambda\right)$ is further simplified as

$$\begin{aligned} F\left( \lambda\right)=\int_{0}^{\infty} {circ}_{0}\left( \rho\right)J_{0}\left( \lambda\rho\right)\rho d\rho=\int_{0}^{1} J_{0}\left( \lambda\rho\right)\rho d\rho=\frac{1}{\lambda}J_{1}\left( \lambda\right)\#\left( \mathrm{SI}.20 \right) \end{aligned}$$

where $J_{1}\left( \lambda\right)$ is a first-order Bessel function. By substituting Equation SI.20 into Equation SI.19, the following equation is got.

$$\begin{aligned} {circ}_{0}\left( \rho\right)=\int_{0}^{\infty} J_{1}\left( \lambda\right)J_{0}\left( \lambda\rho\right)d\lambda\#\left( \mathrm{SI}.21 \right) \end{aligned}$$

Then, the Fourier-Bessel integral of $circ\left( \rho\right)$ before normalization is expressed as

$$\begin{aligned} circ\left( \rho\right)=c_{0}-c_{0}\int_{0}^{\infty} J_{1}\left( \lambda\right)J_{0}\left( \frac{\lambda}{\rho_{0}}\rho\right)d\lambda\#\left( \mathrm{SI}.22 \right) \end{aligned}$$

According to the Equation SI.17, the form of the radial solution is changed from an infinite series to an infinite integral, no longer a linear combination of $J_{0}\left( r \right)$. The integral constant can be regarded as a generalized coefficient of the Fourier-Bessel integral expansion. By comparing with Equation SI.17 and Equation SI.22, we can receive $r=\frac{\lambda}{\rho_{0}}\rho$, *i.e.*

$$\begin{aligned} \sqrt{\frac{E}{D}}=\frac{\lambda}{\rho_{0}}\#\left( \mathrm{SI}.23a \right) \end{aligned}$$

The quantitative relationship can be exhibited between the constant *E* and the diffusion coefficient *D*, *i.e.*

$$\begin{aligned} E=\left( \frac{\lambda}{\rho_{0}} \right)^{2}D\#\left( \mathrm{SI}.23b \right) \end{aligned}$$

**Solution of the final concentration field expressions**

The time equation and the radial equation are considered to obtain the two-dimensional polar diffusion equation under the condition of definite solution.

$$\begin{aligned} c\left( \rho,t \right)=c_{0}-c_{0}\int_{0}^{\infty} J_{1}\left( \lambda\right)J_{0}\left( \frac{\lambda}{\rho_{0}}\rho\right)e^{-\left( \frac{\lambda}{\rho_{0}} \right)^{2}Dt}d\lambda\#\left( \mathrm{SI}.24 \right) \end{aligned}$$

According to $\rho=0$ and $J_{0}\left( 0 \right)=1$, the concentration at the center point is expressed as:

$$\begin{aligned} c\left( 0,t \right)=c_{0}-c_{0}\int_{0}^{\infty} J_{1}\left( \lambda\right)e^{-\left( \frac{\lambda}{\rho_{0}} \right)^{2}Dt}d\lambda\#\left( \mathrm{SI}.25 \right) \end{aligned}$$

When $\lambda_{0}=\frac{\rho_{0}}{\sqrt{Dt}}$, the Equation SI.24 can be further expressed as

$$\begin{aligned} c\left( 0,t \right)=c_{0}-c_{0}\int_{0}^{\infty} J_{1}\left( \lambda\right)e^{-\frac{\lambda^{2}}{\lambda_{0}^{2}}}d\lambda\#\left( \mathrm{SI}.26 \right) \end{aligned}$$

It can be seen that $\kappa=\frac{1}{\lambda_{0}}=\frac{\sqrt{Dt}}{\rho_{0}}$ can be used to characterize the diffusion at different time.

**1 Gelation analysis**

**1.1 Principle and process of gelation**


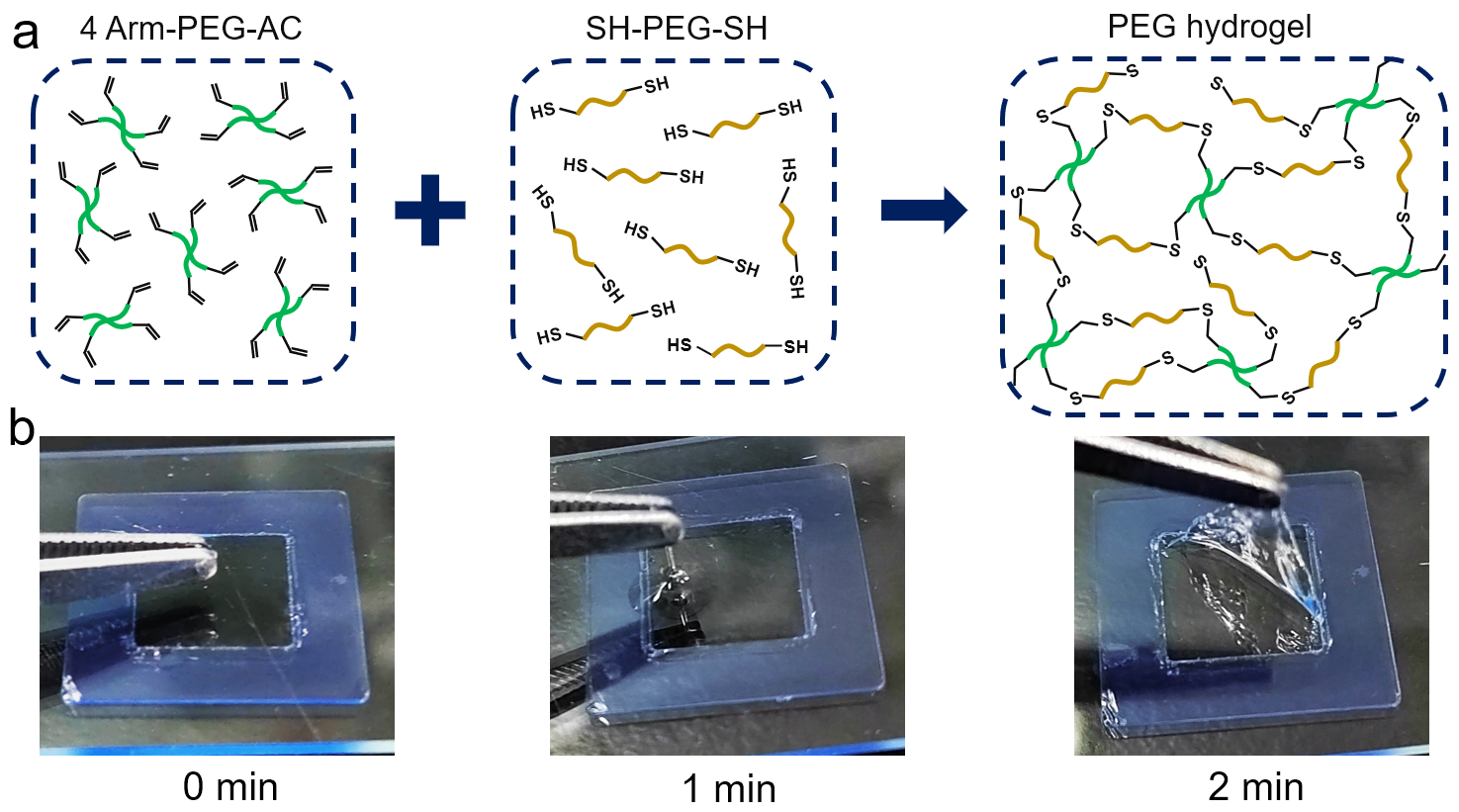


**Figure S1.** (a) Schematic diagram of the formation process of PEG hydrogel by Michael reaction. (b) The gelation process of hydrogels at room temperature.

**1.2 Thermal stability and adhesive strength of hydrogel**

Loss factor of PEG hydrogel is the ratio of the loss modulus and storage modulus, indicating the degree of gelation. During heating for 10 min, this hydrogel has the high elasticity and thermal stability with the low loss factor. Even heating at 65^o^C for 60 min, the flexible hydrogel was still intact (**Figure S2a**). Thus, PEG hydrogel in this work exhibits the excellent thermal stability. As shown in **Figure S2b**, the prepared hydrogels could be directly adhered with different adhesion strengths to various substances including glass, polypropylene, polyethylene, ceramics and steel.

**
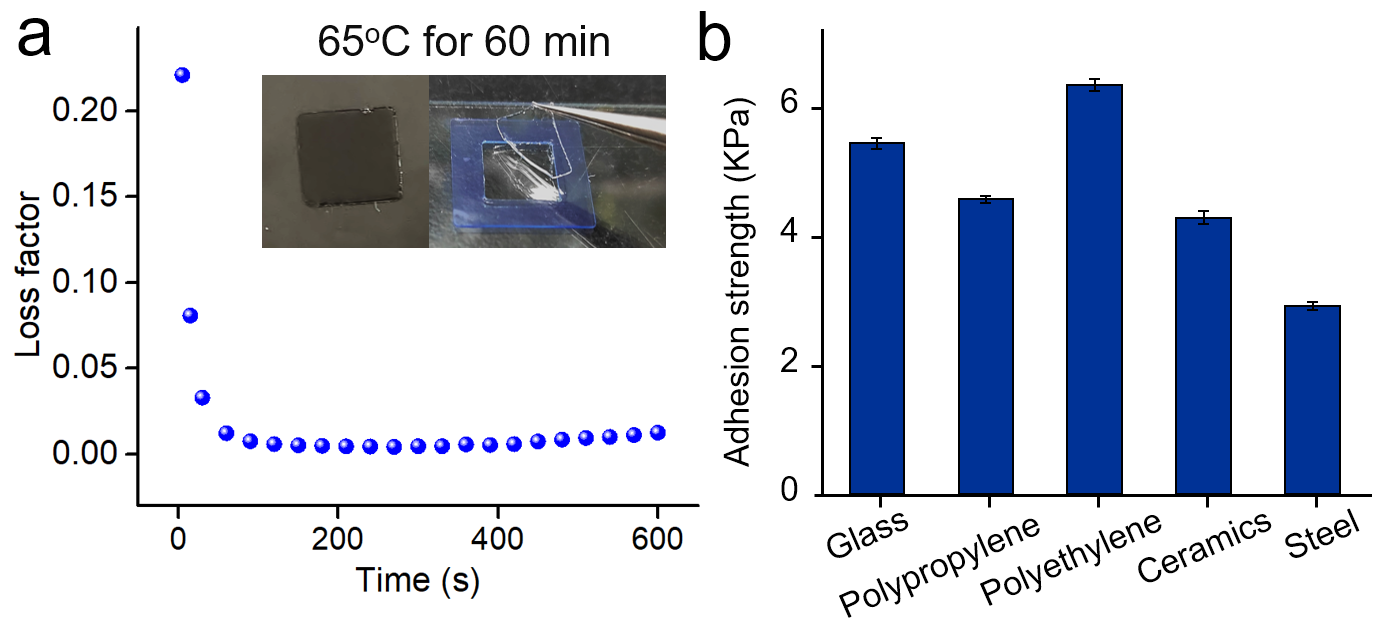
**

**Figure S2.** (a) Loss factor curve of hydrogel over heating time, and photograph of hydrogel after heating at 65^o^C for 60 min. (b) The adhesive strength of PEG hydrogel to glass, polypropylene, polyethylene, ceramics and steel (n=3). Error bars represent the standard deviation from three independent experiments.

**2 Hydrogel iSCIA analysis**

**2.1 Imaging efficiency**

**
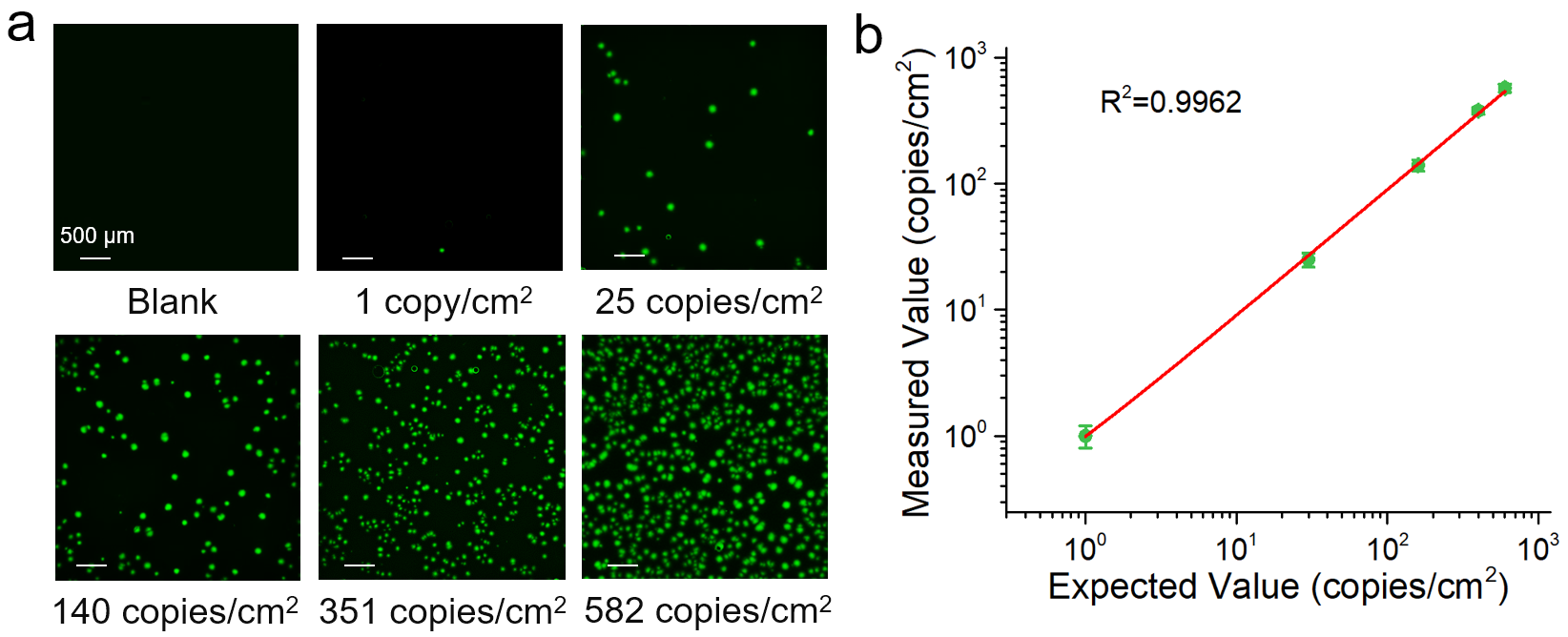
**

**Figure S3.** (a) *In situ* imaging of SARS-CoV-2 RNA with different concentrations after iSCIA. Blank was the negative control without nucleic acid. (b) Linear relationship of hydrogel amplicon dots and target nucleic acids (n=3). A positive correlation was showed between the count of fluorescent dots and the expected number of nucleic acids. Error bars represent the standard deviation from three independent experiments.

Different PEG molecular weight was optimized and discussed. With the increase of molecular weight of 4-Arm PEG acrylate from MW 10000 to MW 20000, the size of amplicon dots became larger, while the number of fluorescent dots was reduced. While the decrease of 4-Arm PEG acrylate from MW 10000 to MW 5000 leaded to the incomplete gelation. With the increase of SH-PEG-SH from MW 3400 to MW 5000, the number of fluorescent dots was slightly reduced. While the decrease of SH-PEG-SH from MW 3400 to MW 2000 leaded to the incomplete gelation. According to the above results, setting 2 with 4-Arm PEG acrylate (MW 10000) and SH-PEG-SH (MW 3400) was selected as optimal molecular weight of monomers (**Figure S4**).


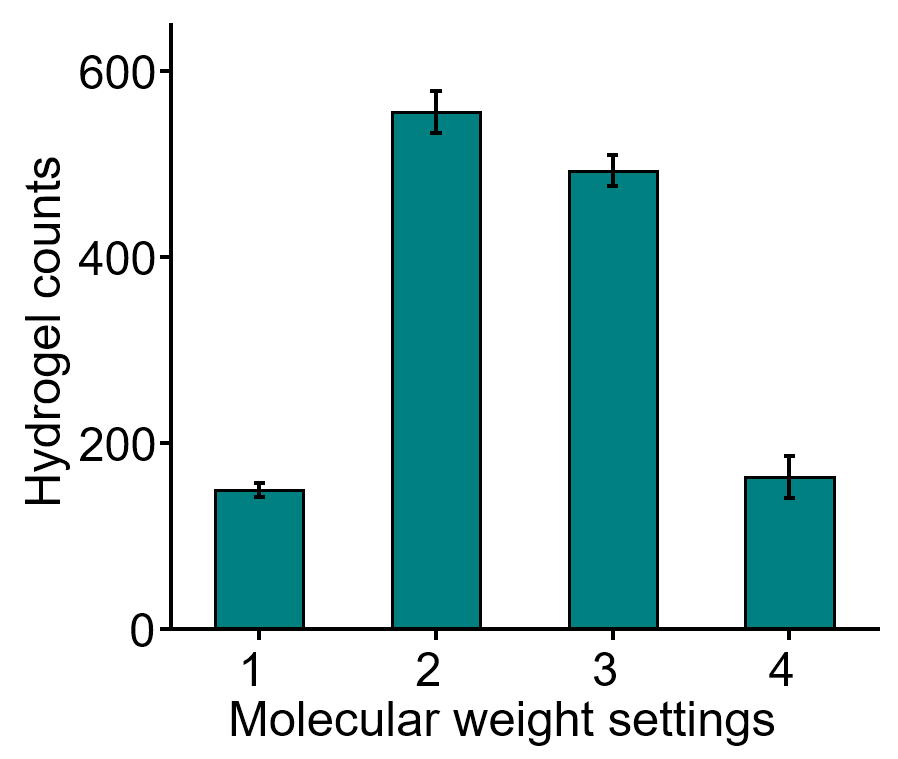


**Figure S4.** Hydrogel counts with different molecular weights of PEG monomer (n=3). Setting 1: Four-arm PEG acrylate (MW 20000) and SH-PEG-SH (MW 3400). Setting 2: Four-arm PEG acrylate (MW 10000) and SH-PEG-SH (MW 3400). Setting 3: Four-arm PEG acrylate (MW 10000) and SH-PEG-SH (MW 5000). Setting 4: Four-arm PEG acrylate (MW 20000) and SH-PEG-SH (MW 5000). Error bars represent the standard deviation from three independent experiments.

**2.2 *In situ* imaging with high concentration of hydrogel and different time**

**
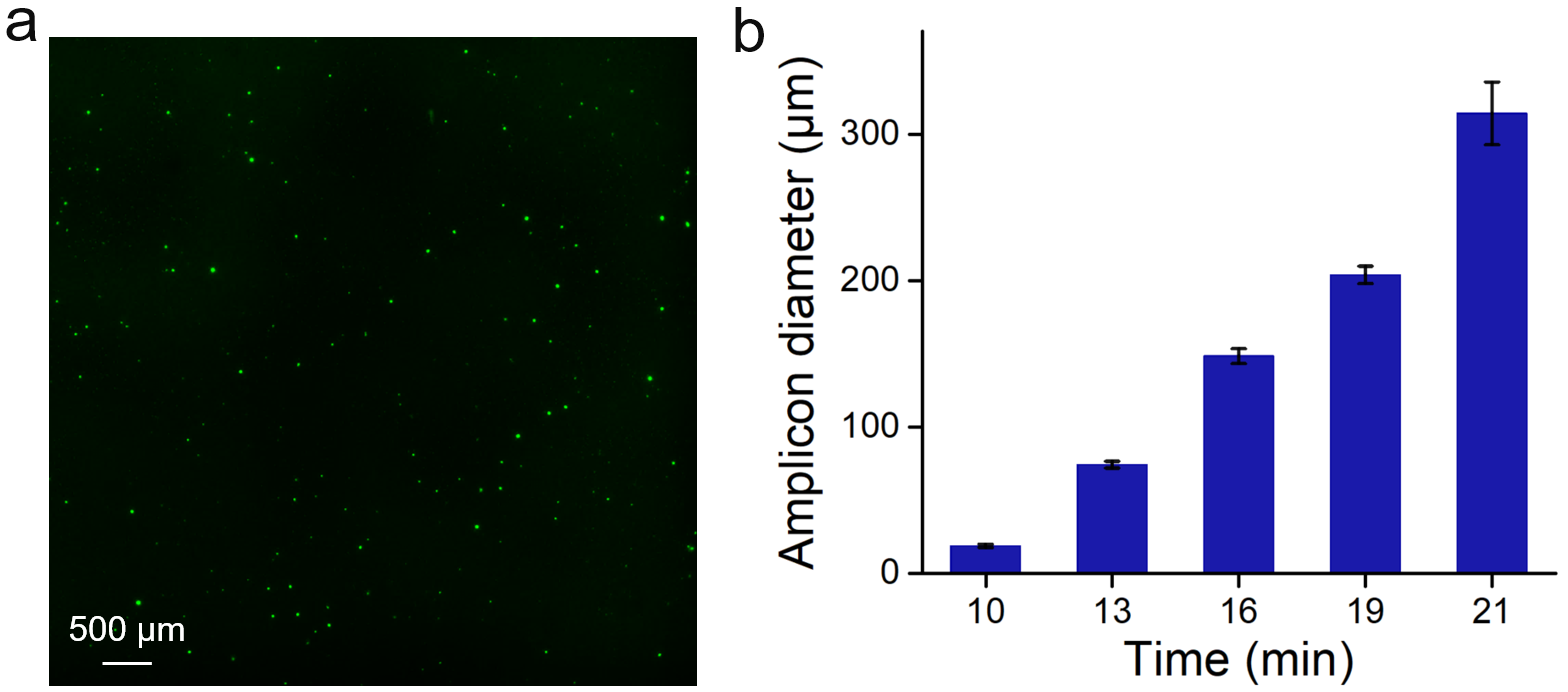
**

**Figure S5.** (a) Fluorescence images of amplicons with high concentration of hydrogel monomers after iSCIA. (b) Amplicon diameters with different heating time for hydrogel iSCIA system (n=3). Error bars represent the standard deviation from three independent experiments.

**2.3 Single-molecule imaging on a large-scale sample**

**
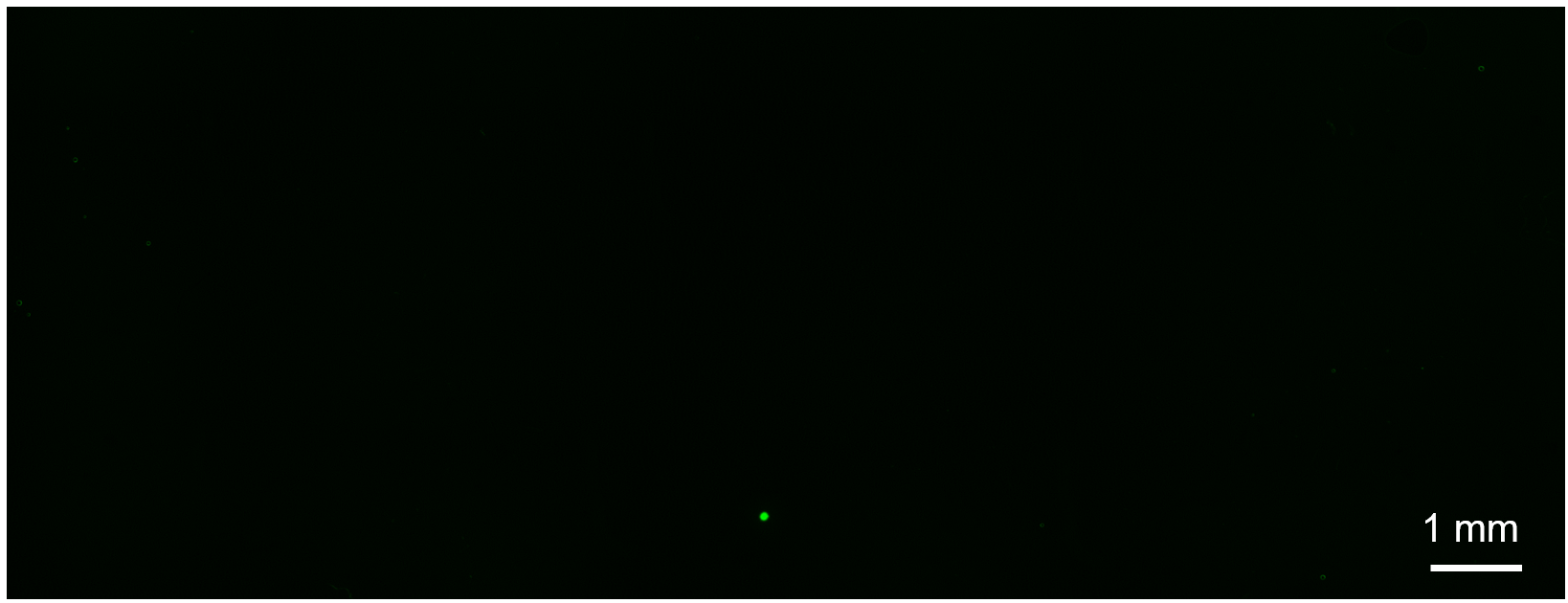
**

**Figure S6.** Fluorescence image of single amplicon on a large-scale sample after hydrogel iSCIA.

**2.4 Boundary of two adjacent amplicon points**

**
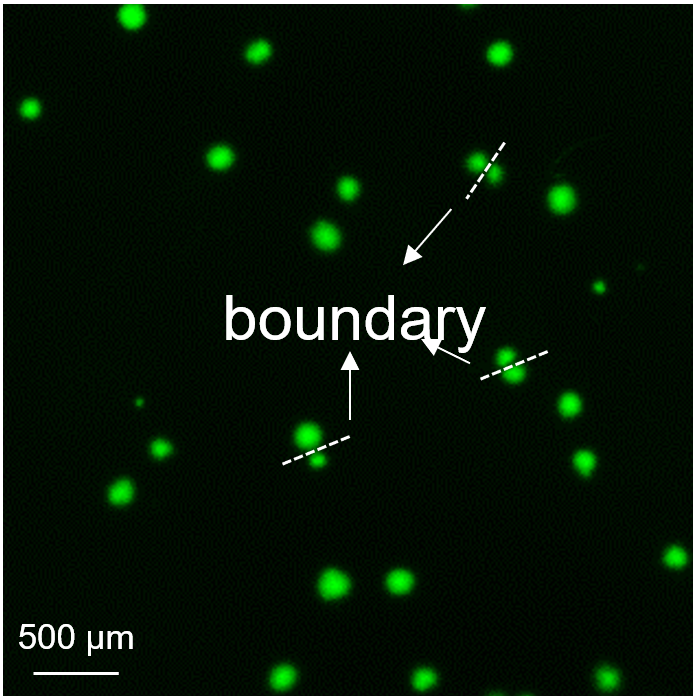
**

**Figure S7.** Fluorescence image with boundary of two adjacent amplicon points after hydrogel iSCIA.

**2.5 Fabrication of PDMS with rough surface**

The spin-coating process and laser direct writing were applied for preparing the SU-8 2075 mould on a silicon wafer. Then, the PDMS with 10:1 of prepolymer and curing agent was covered on the silicon wafer, and cured at 70^o^C for 3 h (**Figure S8a**). The uneven PDMS mould was fabricated with the height of 0.06 mm, the width of 0.8 mm, and the length of 8 mm for each cuboid shape (**Figure S8b**). This PDMS mould was used as a substrate for *in situ* imaging.

**
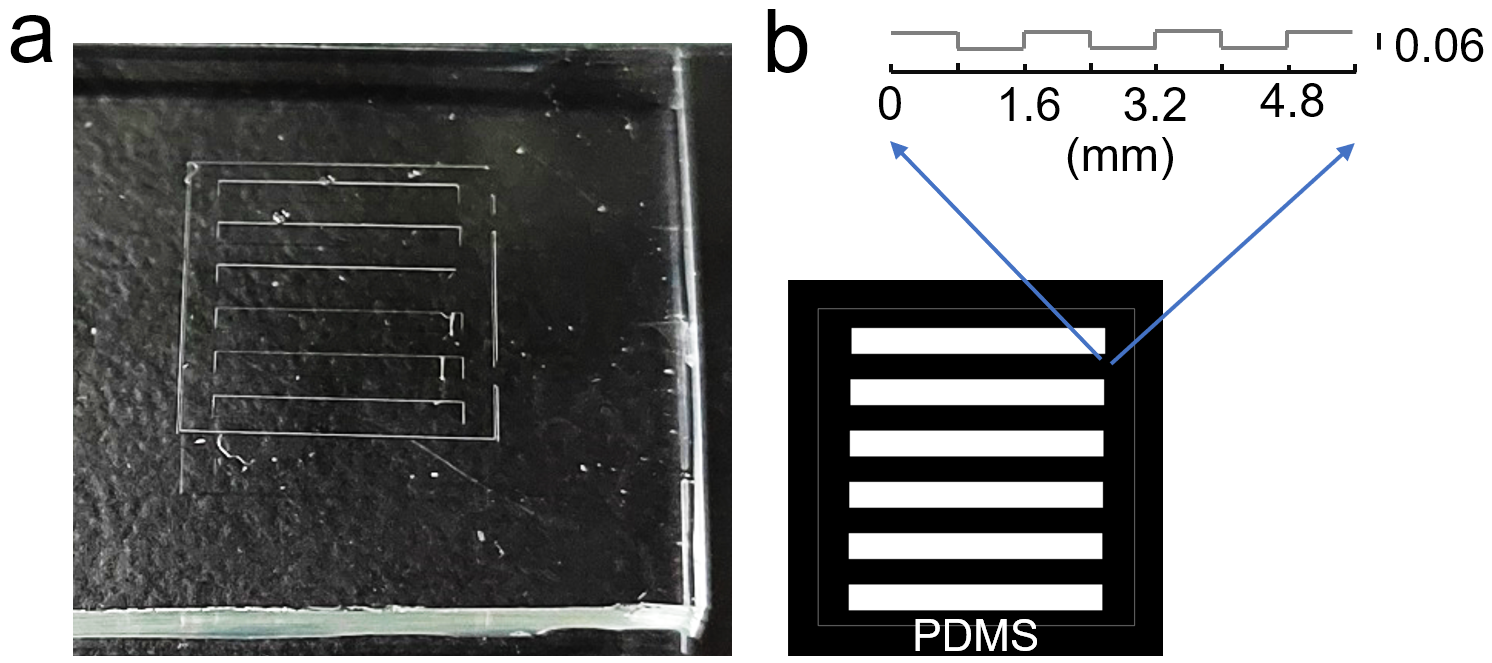
**

**Figure S8.** (a) Photograph of the uneven PDMS mould. (b) Scheme of PDMS mould.

**2.6 *In situ* imaging on porous membrane** **and mouse brain tissue**

**
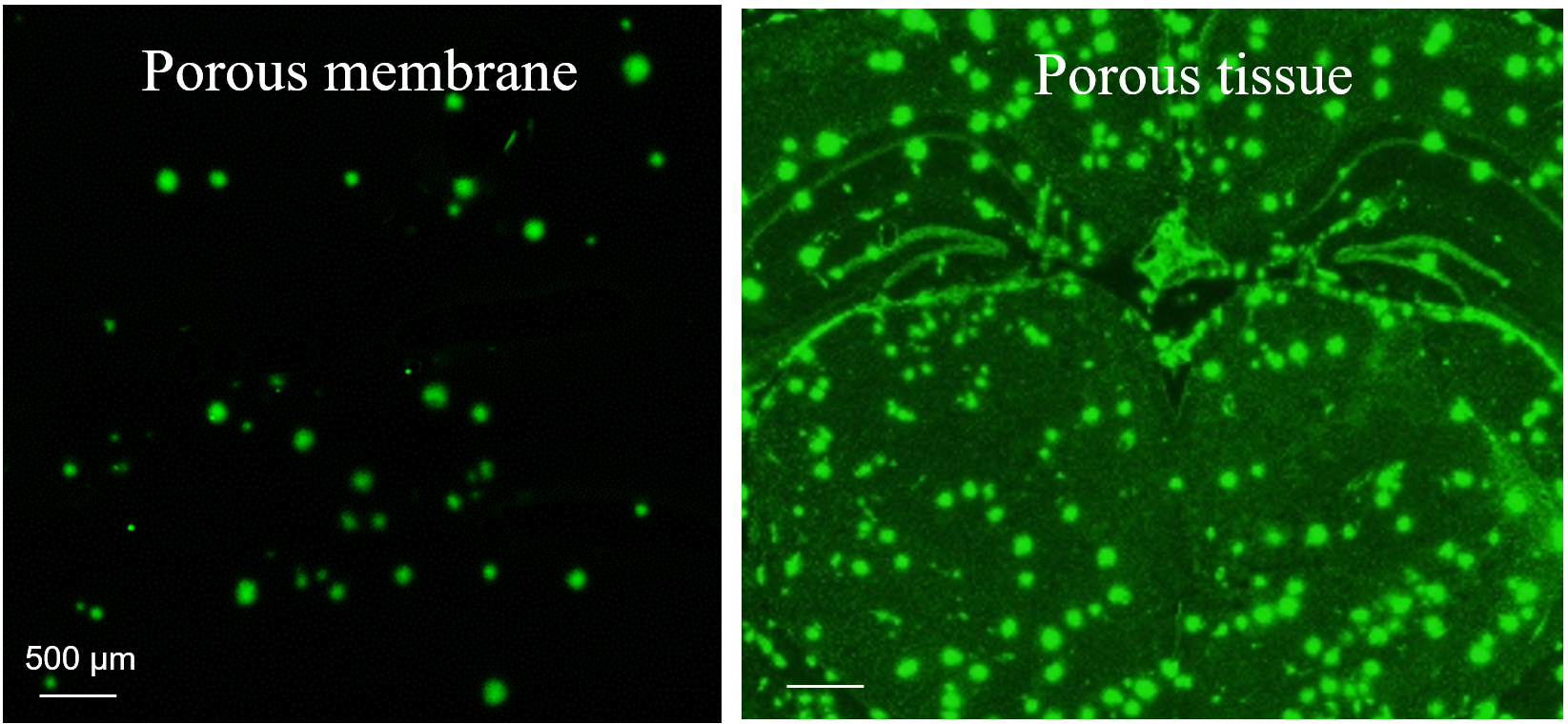
**

**Figure S9.** Fluorescence images of *E. coli* on porous membrane and mouse brain tissue after hydrogel iSCIA.

**2.7 Scanning electron microscope image of hydrogel**


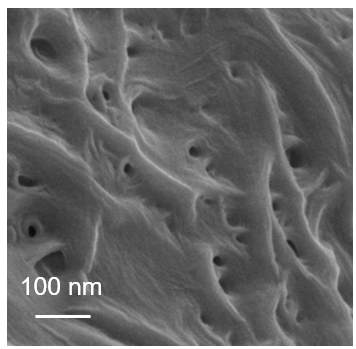


**Figure S10.** Scanning electron microscope image of PEG hydrogel.

The size of hydrogel nanopores can also be calculated by the following classical theory of rubber elasticity related to the shear modulus^[2]^.

$d_{mesh}=\left( \frac{6RT}{\pi N_{Av}G} \right)^{1/3}{10}^{9} (nm)$ (1)

where *R* is the gas constant, *T* is the absolute temperature, *N*_Av_ is the Avogadro’s number, and *G* shear modulus that are tested through the rheometer in Figure 2. The calculated result showed that the hydrogel nanopore was about 20 nm, which was similar to the SEM results.

**3 Confined movement of nucleic acids in hydrogel**

**3.1 Photobleaching experiment**

The fluorescence recovery after photobleaching experiments was used to characterize the nanoconfined movement of nucleic acids inside the hydrogel. The hydrogels loaded with fluorescence-labeled nucleic acids with 43, 150 and 500 nt were exposed to blue light for 10 min, respectively, resulting in a patterned region with weak fluorescence (**Figure S11a**). The fluorescence would be recovered with the diffusion of nucleic acids over time. The diffusion was slower in hydrogel than that in aqueous solution, and the diffusion rate was decreased with the increased nucleic acid lengths (**Figure S11b-c**).

**
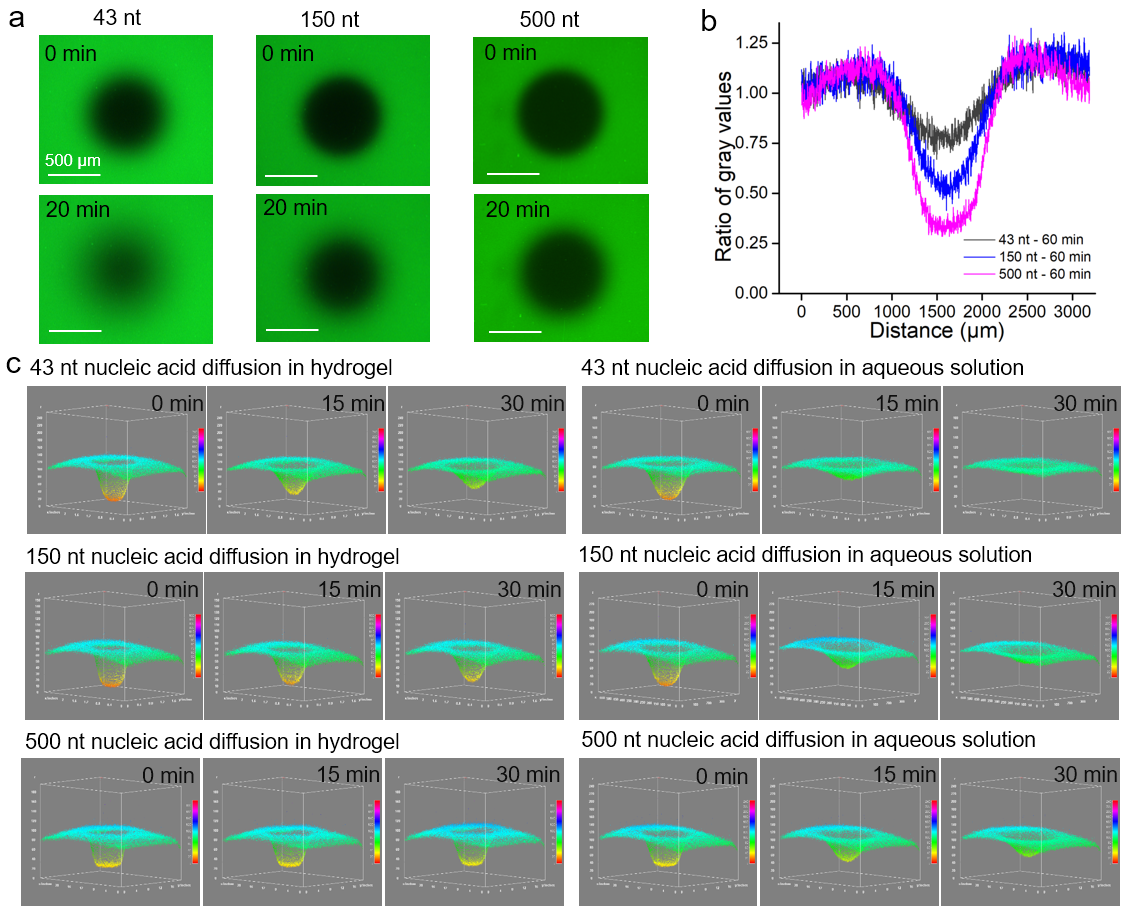
**

**Figure S11.** (a) Fluorescence images of nucleic acid with 43 nt, 150 nt and 500 nt in hydrogel after photobleaching. (b) Corresponding cross-section analysis of fluorescence recovery of nucleic acids with 43 nt, 150 nt and 500 nt in hydrogel at 60 min. (c) Corresponding analysis of fluorescence recovery of nucleic acids with 43 nt, 150 nt and 500 nt in hydrogel and aqueous solution after photobleaching.

**3.2 Comparison of diffusion process**


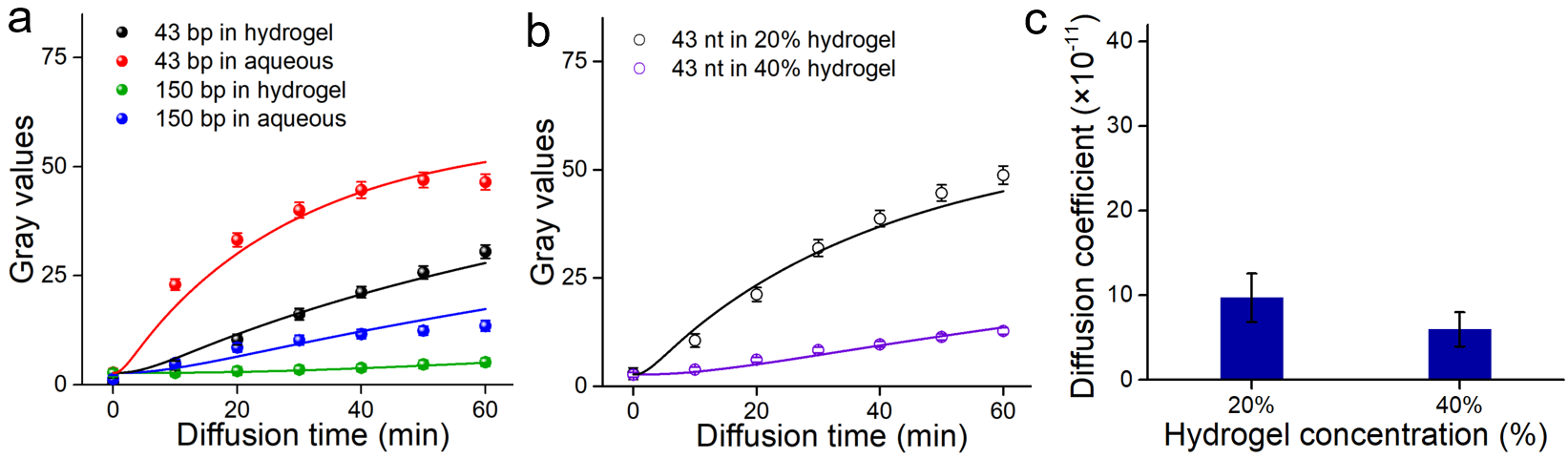


**Figure S12.** (a-b) Fluorescence recovery kinetic curves of nucleic acid with 43 bp and 150 bp in hydrogel and aqueous solution (a), with 43 nt in 20% and 40% hydrogel (b). (c) Diffusion coefficient of nucleic acid with 43 nt in 20% and 40% hydrogel (n=3). Error bars represent the standard deviation from three independent experiments.

**3.3 Patterned molecular imaging**

**
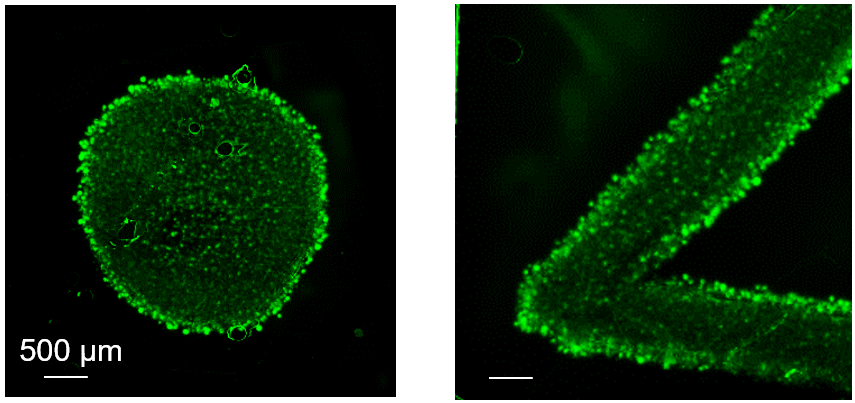
**

**Figure S13.** Fluorescence images of pattened nucleic acids with circular and irregular shapes after hydrogel iSCIA.

**3.4 Naked eye-based observation**

**
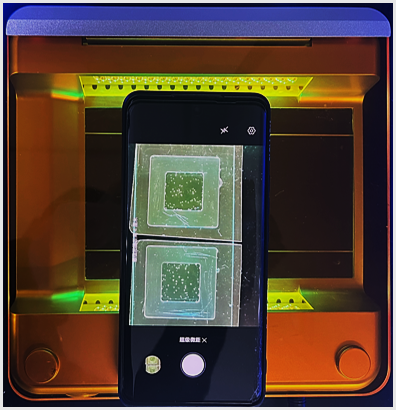
**

**Figure S14.** Photograph of naked eye-based observation with smartphone.

**3.5 Specificity evaluation**

To show the specificity and selectivity of the developed method, *E. coli*, and *L. monocytogenes* were *in situ* imaged. Due to the high specificity of primers, only *E. coli* could be detected (Green dots) by the iSCIA targeting *E. coli*, and only *L. monocytogenes* could be detected (Red dots) by the iSCIA targeting *L. monocytogenes* (**Figure S15a-b**). The selectivity was also verified on similar substrates (polypropylene and polyethylene) and biological sample (lettuce) (**Figure S15c**), revealing the high specificity and selectivity in multiplex settings.


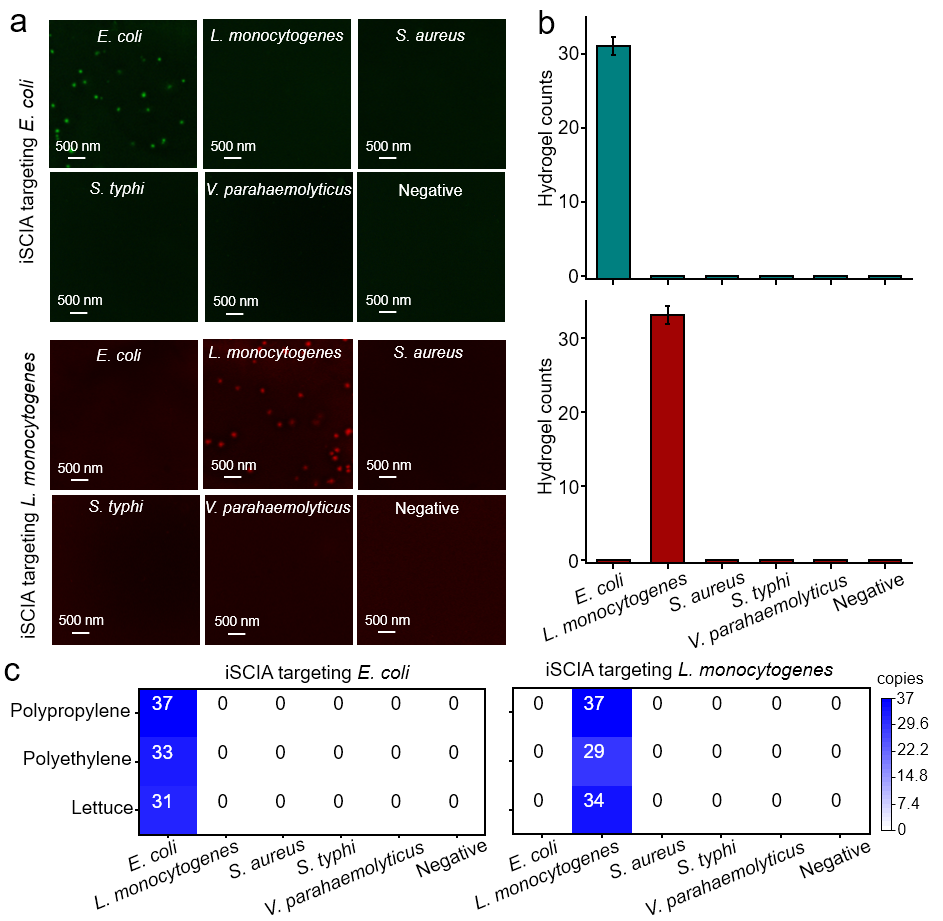


**Figure S15.** The specificity of iSCIA system. (a-b) Fluorescence images (a) and hydrogel counts (b) of iSCIA system for targeting *E. coli* and *L. monocytogenes* (n=3). (c) Specificity of iSCIA on polypropylene, polyethylene and lettuce.

**4 Deep learning for automatically counting**

**4.1 Dataset generation**

The deep learning model was trained using the end-point hydrogel images. Ten images were uploaded to the Roboflow platform and labeled manually for different fluorescent points using the bounding boxes. The annotated bi-colored images were added to the dataset as the training set and validation set. Then, flip, 90^o^ rotation, and crop were used for the augmentation of dataset (**Figure S16a**). The accurate coordinates of different fluorescent amplicons were exported for the follow-up multiplex model training (**Figure S16b**).

**
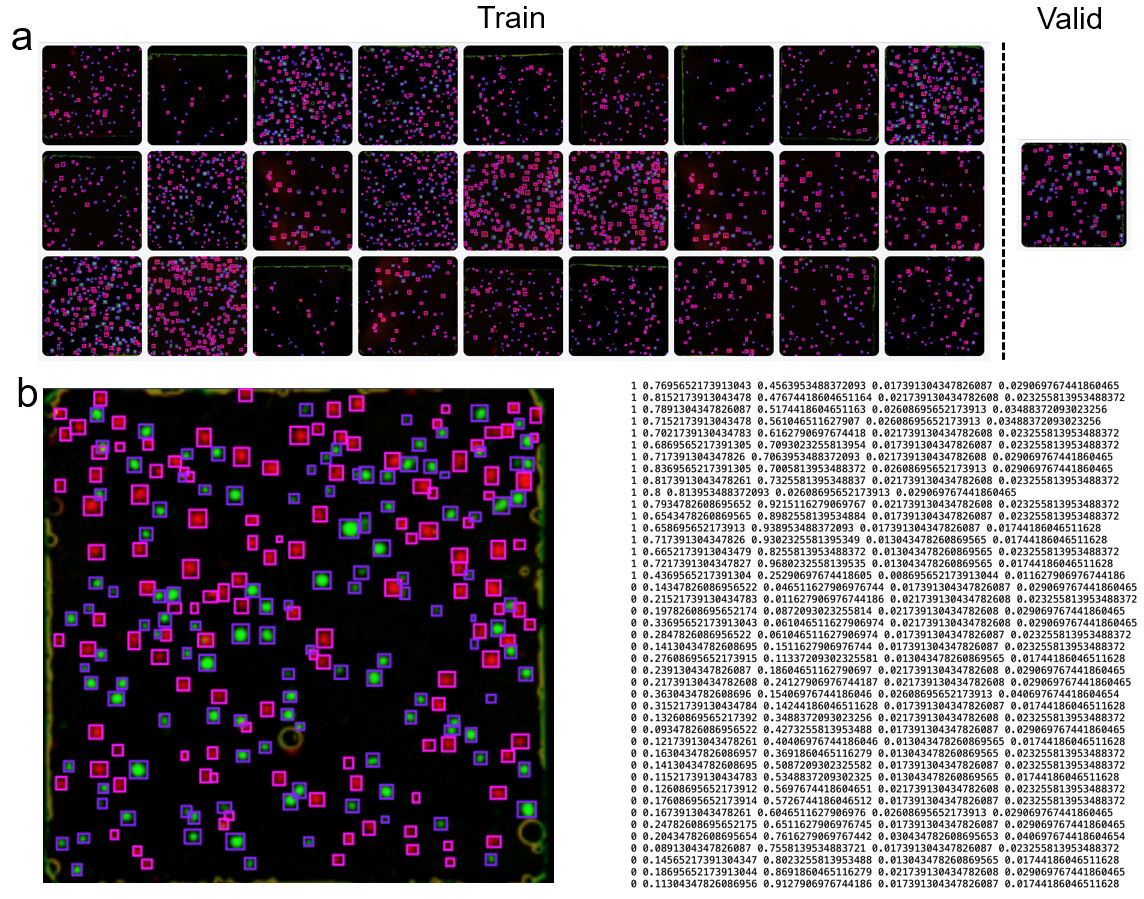
**

**Figure S16.** Dataset generation of hydrogel amplicons. (a) Collected multiplex fluorescence images of amplicons for the training set and validation set. (b) Labelled multiplex amplicons in fluorescence image, and exported coordinates of different amplicons.

**4.2 Performance evaluation**

The YOLOv8m algorithm was used to train the deep learning (DL) model using the collected dataset. The Adam optimizer was selected for training the hydrogel images with small dataset. The number of total epochs was set as 500, while the training would be stopped early in last 50 epochs (patience=50). To improve the model's performance, the hyperparameter and configuration settings, including the batch size and learning rate, were optimized. The sets of hyperparameters and configurations were shown in **Table S1-2**. According to the less epochs and larger mean average precision 50 (mAP 50), the training models with setting 3 and setting 2 were selected as the optimal model for single and multiplex detection model (**Table S1-2**). The initial learning rate and the final learning rate were both set as 0.001 and 0.01, respectively.

In addition, the metric changes of the optimized model in training process were displayed in **Figure S17.** The Area Under Curve (AUC) attained 0.865 and 0.920 in the Precision-Recall (P-R) curve for multiplex and single detection model (**Figure S17a-b**), respectively. During the model training, the loss function curve was declined significantly in 20 epochs, indicating the reduced model losses and rapid model fitting (**Figure S17c-d**). For the multiplex model, train loss and validation loss in the training process were converged after 40 epochs, namely 160 iterations (**Figure S17c**). For the single detection model, as shown in **Figure S17d**, after 50 epochs that were 200 iterations, the train loss and validation loss became converged gradually. These evaluations above provided the excellent performance in the model fitting and generalization, which were high accuracy with the optimized setting.

**
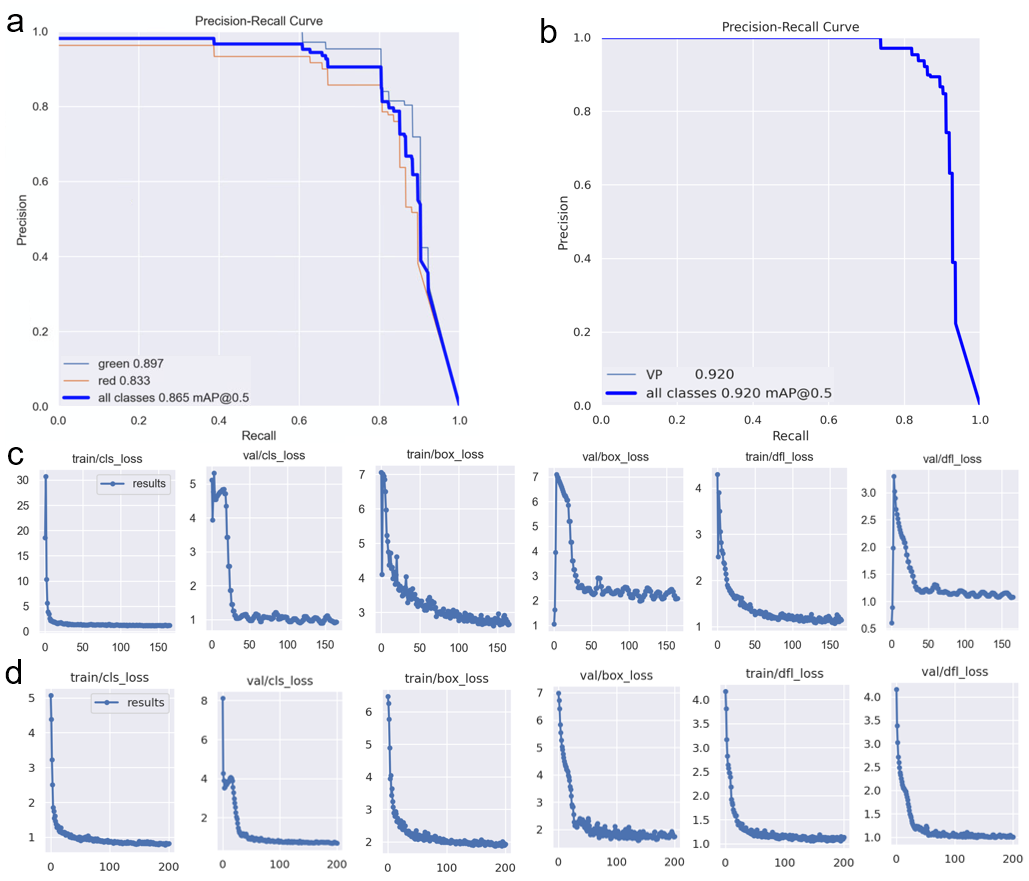
**

**Figure S17.** Performance evaluation for the best deep learning model. (a-b) Precision-Recall (P-R) curve for multiplex (a) and single (b) detection model. Mean Average Precision (mAP) was the Area Under Curve (AUC). (c-d) Train loss and validation loss in the training process for the multiplex (c) and single (d) detection model.

**Table S1.** Comparison of different hyperparameters and configurations used to train the model for single detection.

| Setting number | Model | Batch size | lr0 | lrf | Best epoch | mAP50 |
| --- | --- | --- | --- | --- | --- | --- |
| 1 | YOLOv8m | 16 | 0.01 | 0.01 | 205 | 0.886 |
| 2 | YOLOv8m | 16 | 0.005 | 0.01 | 177 | 0.902 |
| 3 | YOLOv8m | 16 | 0.001 | 0.01 | 200 | 0.920 |
| 4 | YOLOv8m | 16 | 0.0005 | 0.01 | 200 | 0.911 |
| 5 | YOLOv8m | 16 | 0.0001 | 0.01 | 214 | 0.865 |
| 6 | YOLOv8m | 8 | 0.001 | 0.01 | 230 | 0.913 |

**Table S2.** Comparison of different hyperparameters and configurations used to train the model for multiplex detection.

| Setting number | Model | Batch size | lr0 | lrf | Best epoch | Green  AP | Red  AP | mAP50 |
| --- | --- | --- | --- | --- | --- | --- | --- | --- |
| 1 | YOLOv8m | 8 | 0.01 | 0.01 | 179 | 0.780 | 0.833 | 0.807 |
| 2 | YOLOv8m | 8 | 0.001 | 0.01 | 165 | 0.897 | 0.833 | 0.865 |
| 3 | YOLOv8m | 8 | 0.0001 | 0.01 | 193 | 0.740 | 0.736 | 0.738 |

**4.3 Comparison of counting process**


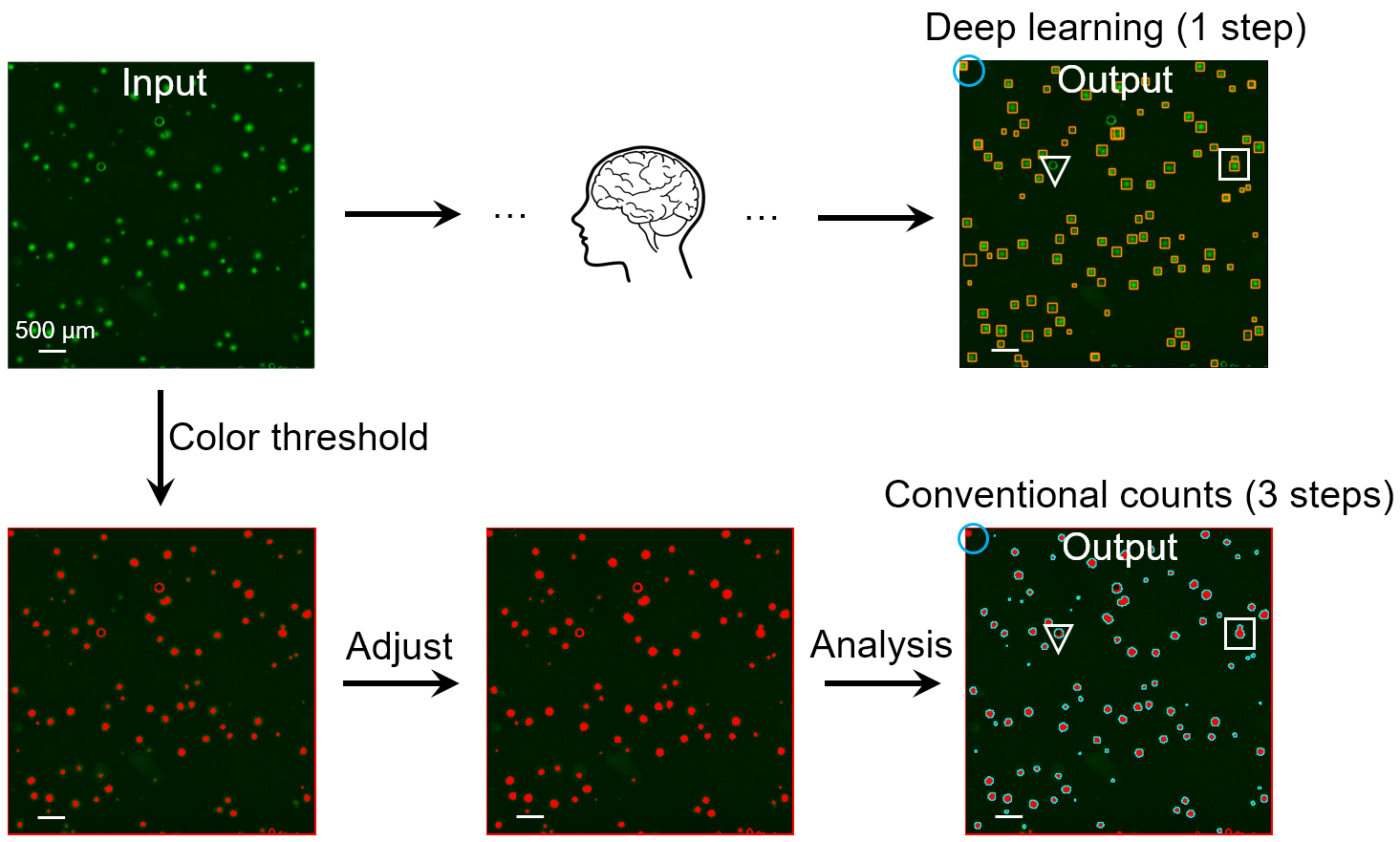


**Figure S18.** Schematic diagram of counting process for hydrogel amplicons using deep learning and conventional software.


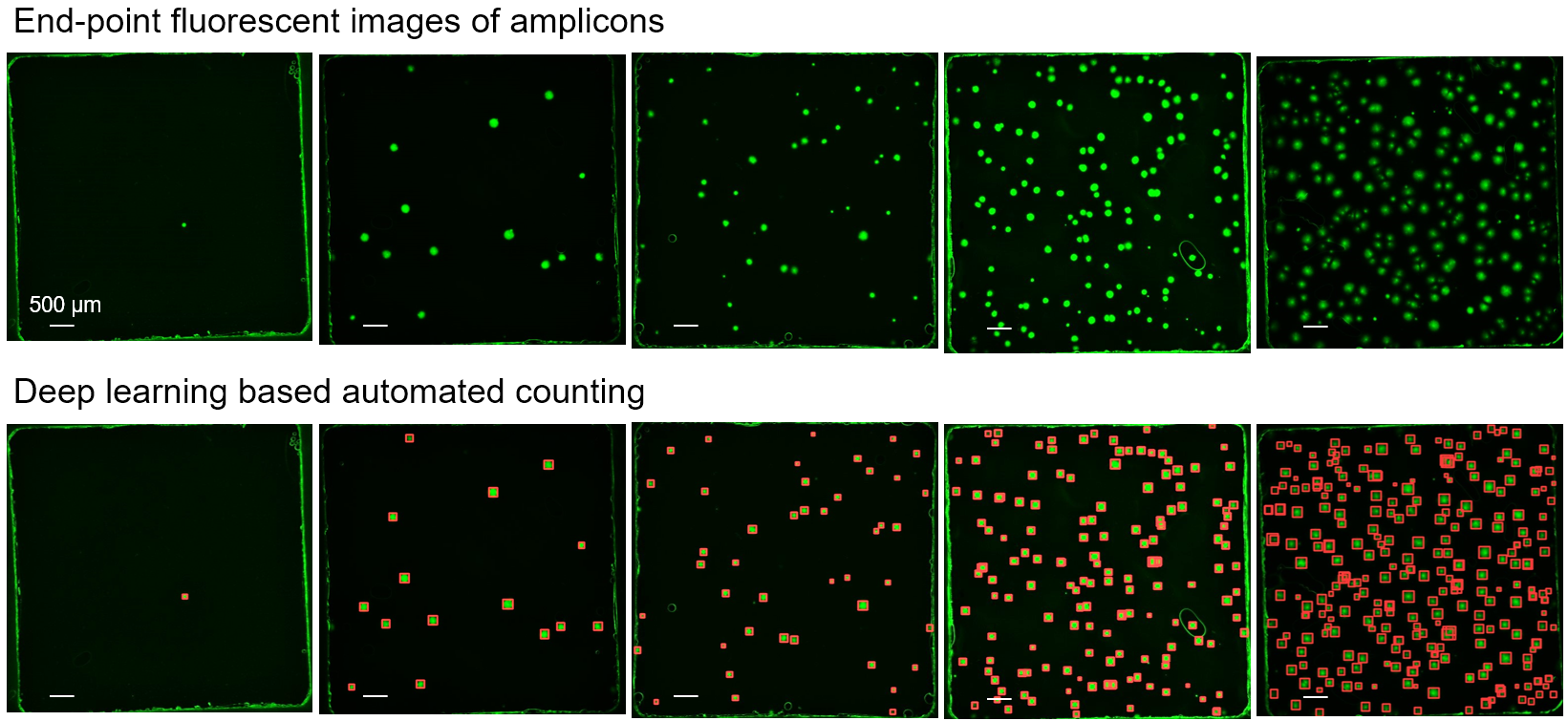


**Figure S19.** Fluorescence images with different amplicon concentrations and automated counts using deep learning.

**5 Performance of hydrogel iSCIA**

**5.1 Cross-sectional shape of amplicon on various materials**


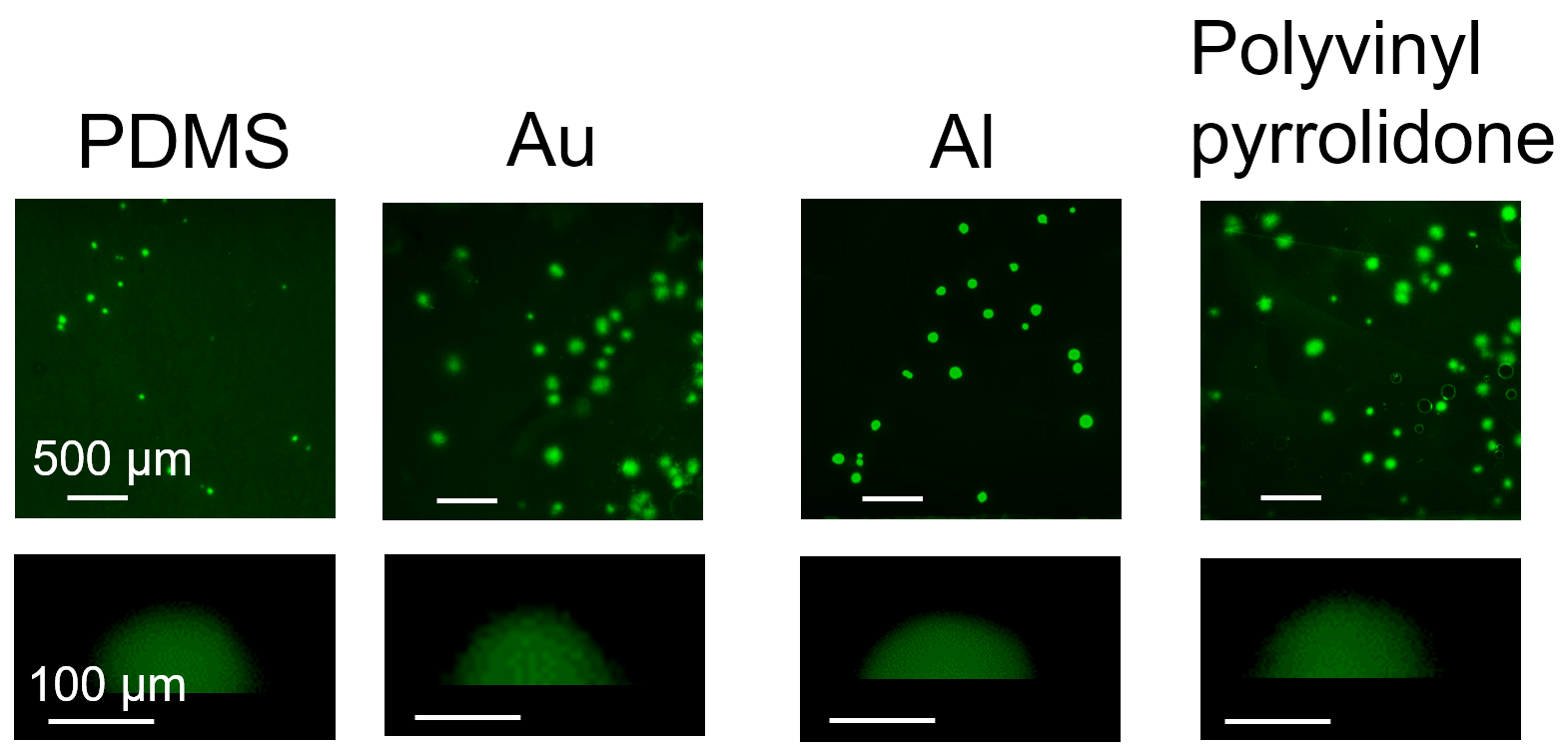


**Figure S20.** Top-view images and cross sectional-view LSCM images of SARS-CoV-2 amplicons after iSCIA on various materials.

**5.2 Reproducibility** **across different samples**


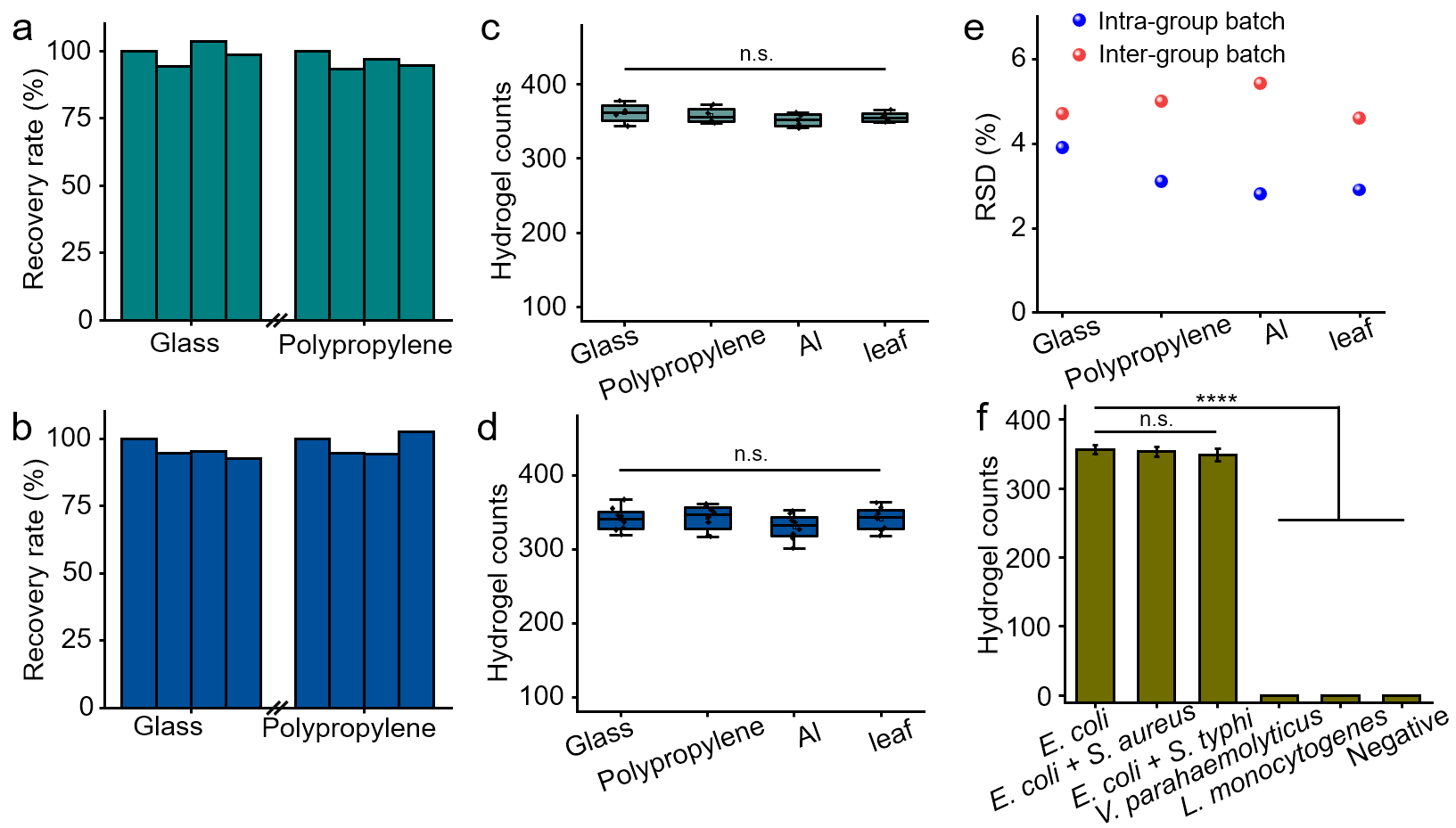


**Figure S21.** The reproducibility of iSCIA across different samples. (a-b) Recovery rate of intra-group batch (a) and inter-group batch (b) on glass and polypropylene. (c-d) Hydrogel counts of intra-group batch (c) and inter-group batch (d) on glass, polypropylene, Al and leaf (n=3). (e) Relative standard deviation of iSCIA imaging. (f) Hydrogel counts with different bacteria on glass (n=3). The statistical signiﬁcance was determined using a two-tailed unpaired Student’s t-test. Statistically signiﬁcant results were indicated in the ﬁgures using *****p* < 0.0001, and no signiﬁcance (n.s., *p* > 0.05).

**5.3 Stability of iSCIA** **in different conditions**

The high stability of hydrogel system could be obtained within 60^o^C-70^o^C (**Figure S23a**), suggesting the tolerance for reaction temperature. Besides, the high stability of hydrogel system was also revealed for sample with wide pH levels (pH 1 - pH 11) (**Figure S23b**). And the anti-interference performance of our iSCIA system was demonstrated by adding various competing molecules in nucleic acid amplification, including humic acid, hemoglobin and anthocyanin (**Figure S23c**). The presence of these competing molecules could not affect the hydrogel iSCIA results.


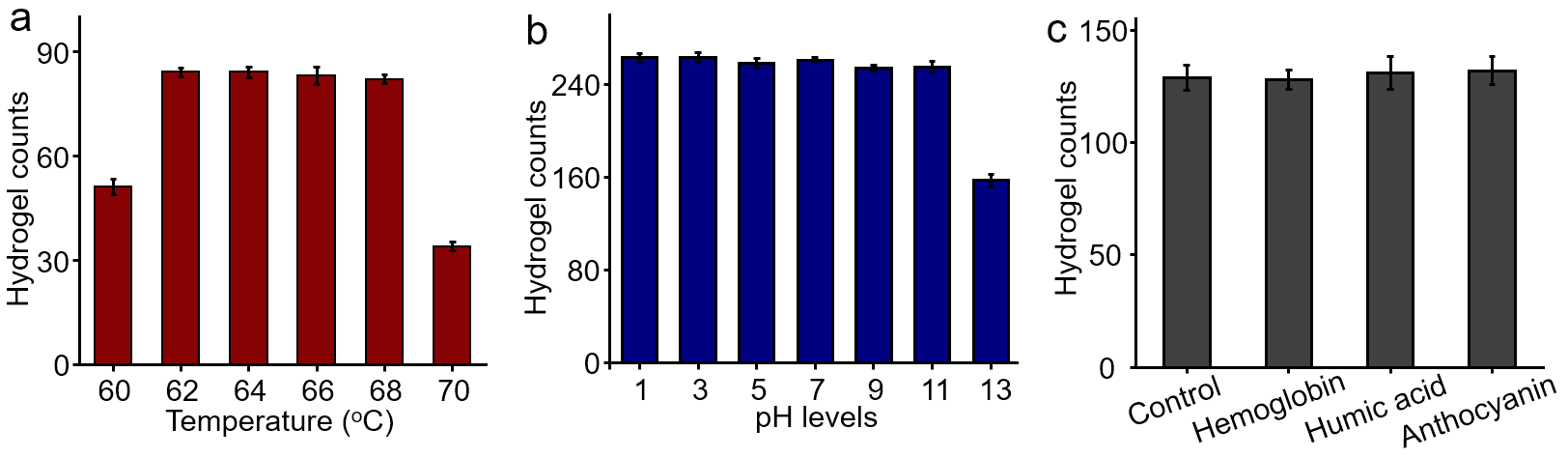


**Figure S22.** (a-c) The stability of iSCIA under varying temperatures (a), pH levels (b) and competing molecules (c) (n=3). Error bars represent the standard deviation from three independent experiments.

**5.4 Long-term stability after storage and transportation**


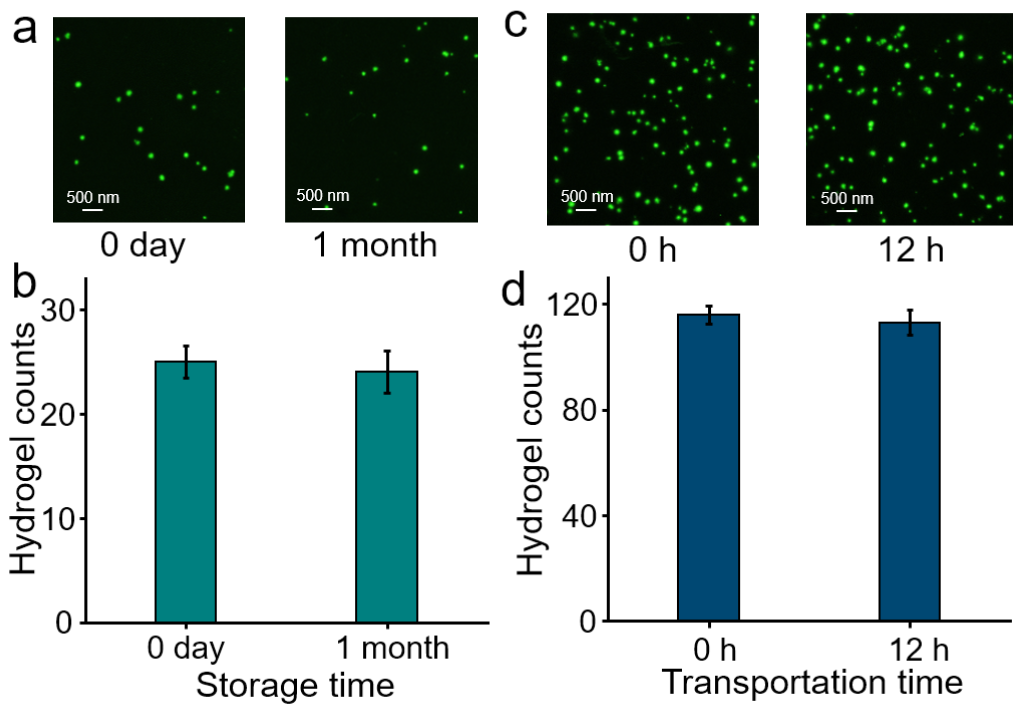


**Figure S23.** Long-term stability of iSCIA system. (a-b) Fluorescence image (a) and hydrogel counts (b) of iSCIA system before and after storage at 4^o^C (n=3). (c-d) Fluorescence image (c) and hydrogel counts (d) of iSCIA system before and after transportation (n=3). Error bars represent the standard deviation from three independent experiments.

**5.5 *In situ* imaging of pathogens on diverse real samples**


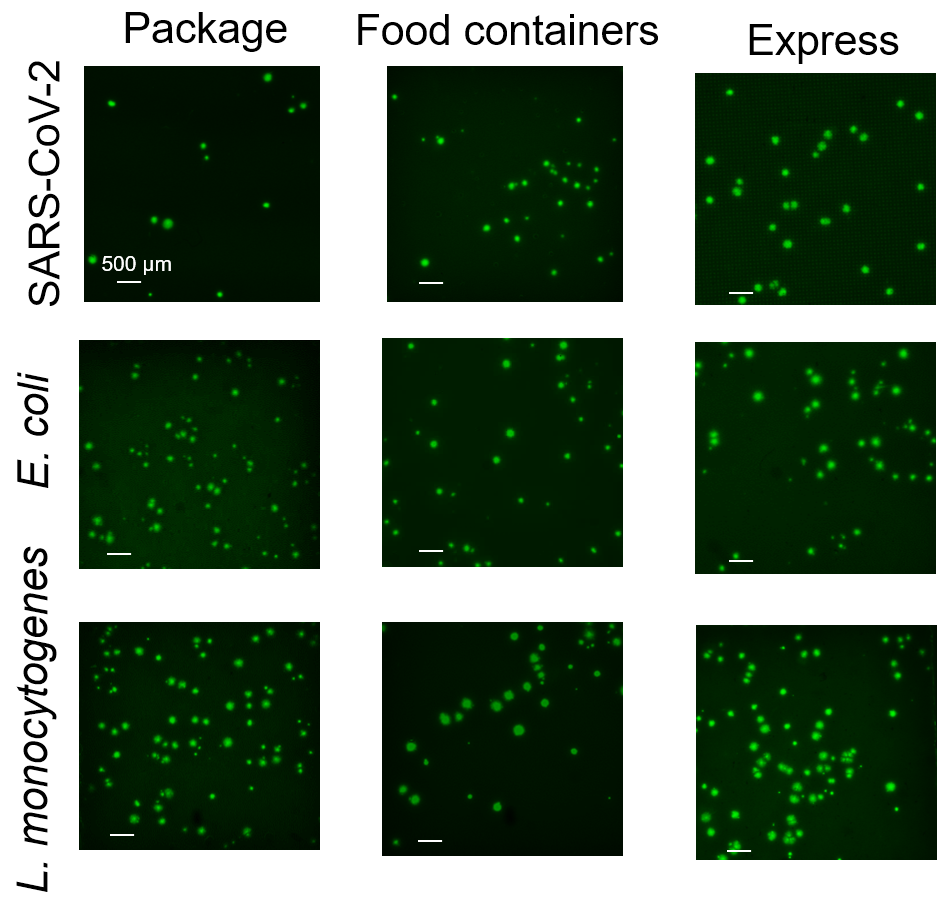


**Figure S24.** Fluorescence images of SARS-CoV-2, *E. coli* and *L. monocytogenes* on diverse real samples after hydrogel iSCIA.

**6 *In situ* imaging on biological samples**

**6.1 Hydrogel peeling-off iSCIA**

The peeling-off iSCIA assay was prepared to construct the universal *in situ* imaging system on samples (**Figure S25a**). The prepared iSCIA hydrogel was first covered on plant samples. Then, the adhesive hydrogel was peeled off, and incubated at 65°C for subsequent isothermal amplification. Amplicon dots were observed without targets loss on the hydrogel under the blue light (**Figure S25b**), showing the location of target molecules. In this case, the self-fluorescence background of plant samples could be eliminated, and the plant health could be monitored without damage by the attached hydrogel (**Figure S25c**).

**
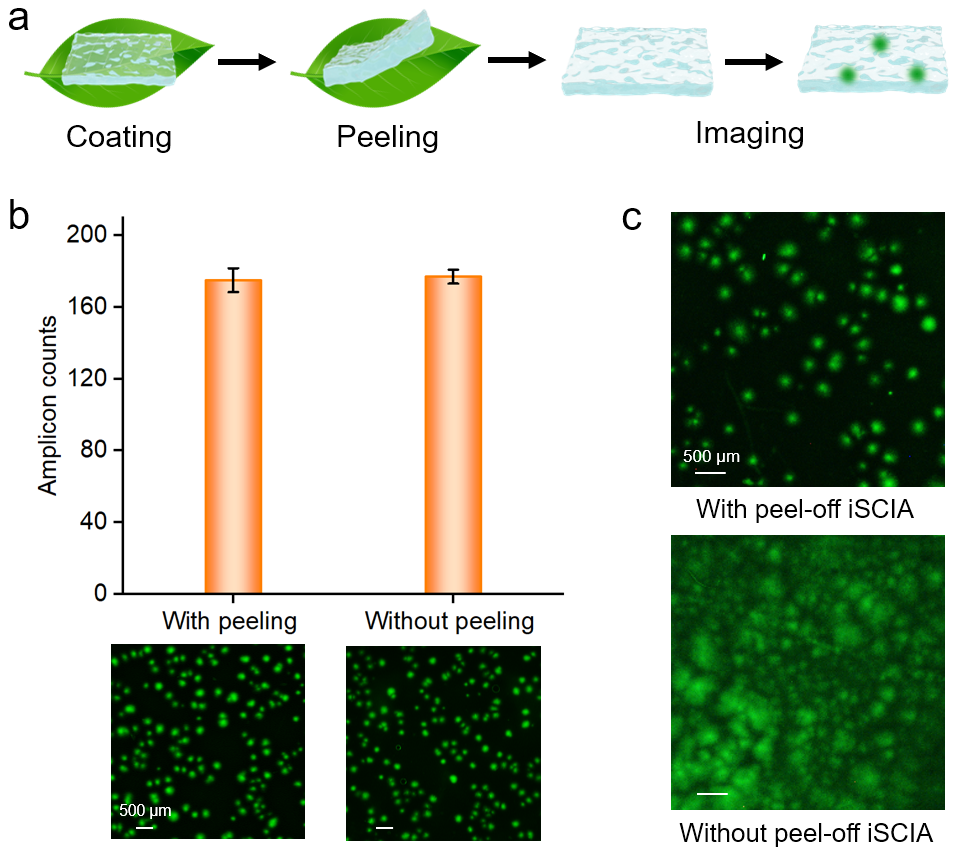
**

**Figure S25.** (a) Schematic diagram of hydrogel peeling-off iSCIA process on plant leaf. (b) Amplicon counts using direct iSCIA and peeling-off iSCIA (n=3). Pathogens on the sample could be imaged without targets loss. Error bars represent the standard deviation from three independent experiments. (c) Fluorescence images using direct iSCIA and peeling-off iSCIA on grape. The high fluorescence background could be eliminated by peel-off iSCIA.

**6.2 *In situ* imaging of *E. coli* O157:H7 infection on lettuce**

**
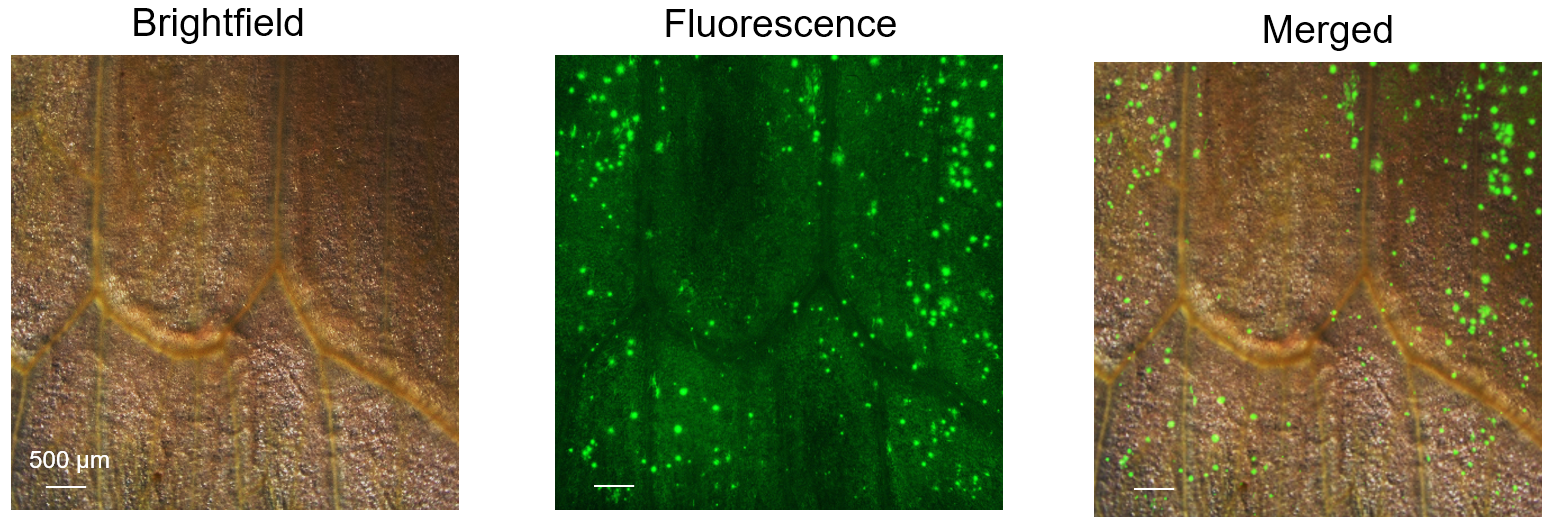
**

**Figure S26.** Bright-field, fluorescence and merged images of *E. coli* O157:H7 on the lettuce.

**6.3 *In situ* imaging of *E. coli* on Green Tea**

**
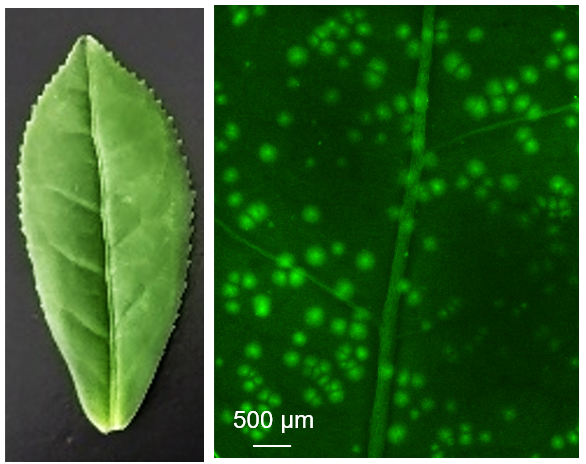
**

**Figure S27.** Photograph of Green Tea (left), and fluorescence images of *E. coli* on Green Tea after hydrogel iSCIA (right).

**6.4 *In situ* imaging of SARS-CoV-2 on cold-chain fruits**

**
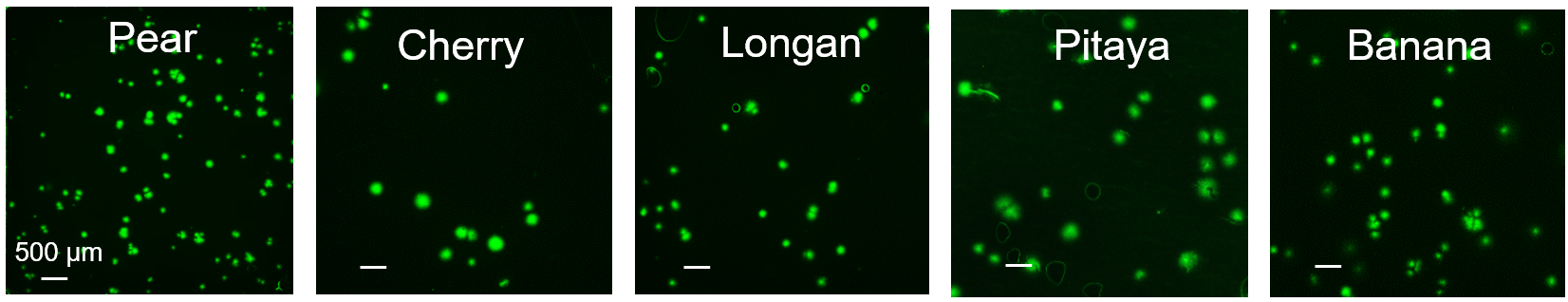
**

**Figure S28.** Fluorescence images of SARS-CoV-2 after hydrogel iSCIA on various fruits.

**6.5 Practical detection from environmental samples**


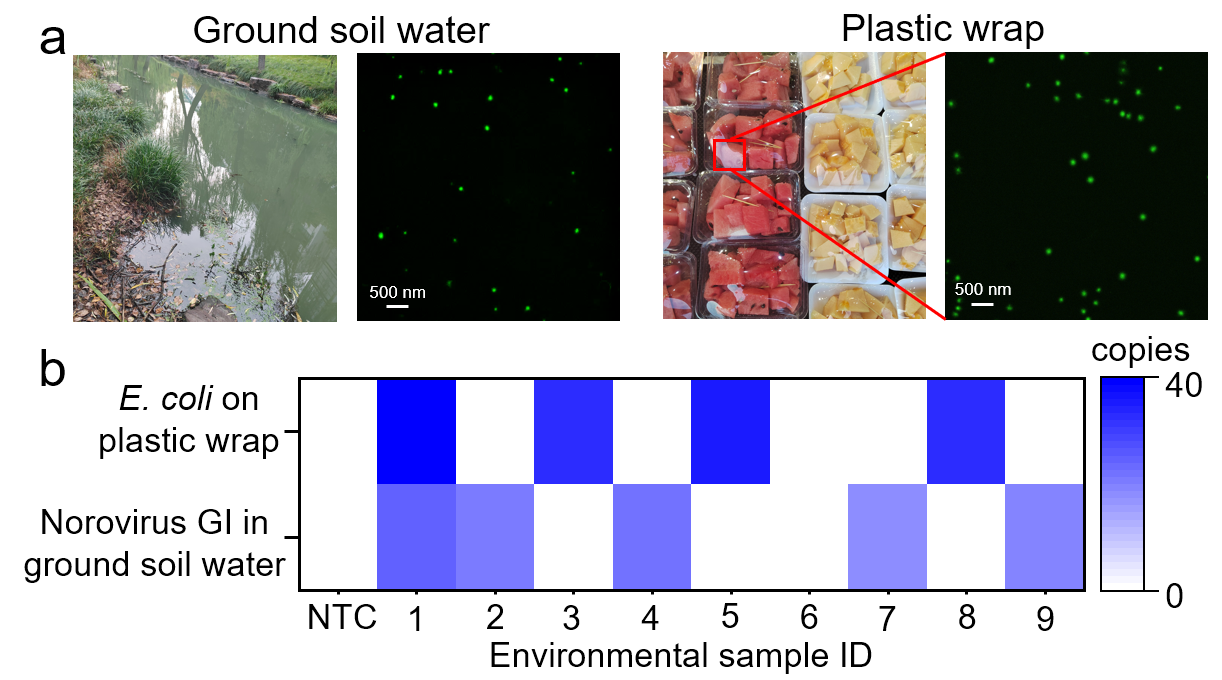


**Figure S29.** *in situ* imaging of actual environmental sample with naturally occurring Norovirus GI on ground soil water and *E. coli* on the plastic wrap that covering fresh food in the supermarket. (a) fluorescence image. (b) hydrogel counts. NTC: No Template Control.

**Table S3** Comparation of our iSCIA technique and existing *in situ* imaging methods

| Methods | Pre-fixation | Detection time | Mild condition | Imaging efficiency | Cost | Ease of use | Direct Real sample analysis |
| --- | --- | --- | --- | --- | --- | --- | --- |
| iSCIA | No | < 30 min | Yes | High | $0.85 | Yes | Yes |
| FISH | Yes | > 14 h | Yes | Medium | $40.4 | No | No |
| *In situ* RCA | Yes | > 10 h | Yes | Medium | $4.52 | No | No |
| *In situ* PCR | Yes | > 10 h | No | Medium | $2.13 | No | No |
| *In situ* LAMP | Yes | > 10 h | Yes | Medium | $2.71 | No | No |

Note: iSCIA: *in situ* space-confined interfacial amplification. FISH: fluorescence *in situ* hybridization. RCA: rolling circle amplification. PCR: polymerase chain reaction. LAMP: loop-mediated isothermal amplification.

**7 Sequences of primers**

**Table S4** Sequences of primers for iSCIA amplification

| Targets | Name | Sequences (5' to 3') |
| --- | --- | --- |
| SARS-CoV-2 | F3 | GTTCCTCATCACGTAGTCG |
|  | B3 | GTTTGGCCTTGTTGTTGTT |
|  | FIP | GCCAGCCATTCTAGCAGGAGCAACAGTTAAGAAATTCAACTCC |
|  | BIP | GATGCTGCTCTTGCTTTGCTACCAGACATTTTGCTCTCAA |
|  | LB | GCTGCTTGACAGATTGAACCAG |
| Norovirus | F3 | TTCCATGACCTCGGATTGTG |
|  | B3 | GCGACTGCTGTCGAAGAAC |
|  | FIP | CCACGCTTGATGTAGCGTCCTTGGAGATCGCGATCTTCTGC |
|  | BIP | TAGTGGCGCTGGTCAGTTGGCTGCTACAGGATCCATTGCA |
|  | LF | CGCCATCATCATTTACGAATTCGG |
|  | LB | TACCGGAGGTTAATGCTTCTGA |
| *Alternaria alternata* | F3 | AAGATCACTGTCAAGGGCG |
|  | B3 | ATGGTAAGACCATCGCAGC |
|  | FIP | TGGGCTTGGTCTTTCCACCATTCCGAGGGATCTGTTCTCAAC |
|  | BIP | TTCTCCGCTCACAAACTGACCGAACGACTTGGACGGGAGG |
|  | LF | ACCAACGAGCACCATCACC |
|  | LB | ACTCCACCATCACCGGCAT |
| *Escherichia coli* | F3 | GCCATCTCCTGATGACGC |
|  | B3 | ATTTACCGCAGCCAGACG |
|  | FIP | CATTTTGCAGCTGTACGCTCGCAGCCCATCATGAATGTTGCT |
|  | BIP | CTGGGGCGAGGTCGTGGTATTCCGACAAACACCACGAATT |
|  | LF | CTTTGTAACAACCTGTCATCGACA |
|  | LB | ATCAATCTCGATATCCATGAAGGTG |
|  | FAM-FIP | 6-FAM-CATTTTGCAGCTGTACGCTCGCAGCCCATCATGAATGTTGCT |
|  | Probe-cFIP | GAGCGTACAGCTGCAAAATG-TAMRA |
| *Listeria monocytogenes* | F3 | AAGCTGCTTTTGATGCTG |
|  | B3 | TCGATTAAAAGTAGCGCCTT |
|  | FIP | CGGCTTTGAAGGAAGAATTTTTGATCGTAAGCGGAAAATCTGT |
|  | BIP | TACGGAGGTTCCGCAAAAGATTTTCAAAATATCGCGTAAGTCT |
|  | LB | TGAAGTTCAAATCATCGACGGC |
|  | ROX-FIP | ROX-CGGCTTTGAAGGAAGAATTTTTGATCGTAAGCGGAAAATCTGTCT |
|  | Probe-cFIP | GATCAAAAATTCTTCCTTCAAAGCCG-BHQ2 |

**References**

[1] a) A. Paul, T. Laurila, V. Vuorinen, S. V. Divinski, in *Thermodynamics, Diffusion and the Kirkendall Effect in Solids*, Springer International Publishing, Cham **2014**, p. 115; b) B. Cantor, in *The Equations of Materials*, Oxford University Press, **2020**, 141-161.

[2] J. Y. Li, D. J. Mooney, *Nature Reviews Materials* **2016**, 1, 16071.
